# Palladium(II) Complexes with Noncovalent Interactions with DNA: Solution Speciation Controlled by Solvent Identity, pH, and Concentration

**DOI:** 10.1021/acs.inorgchem.5c04027

**Published:** 2025-11-12

**Authors:** David Fabra, János P. Mészáros, Ana I. Matesanz, Gabriella Spengler, Francisco Aguilar Rico, Guillermo Moreno-Alcántar, Angela Casini, Éva A. Enyedy, Adoración Gómez Quiroga

**Affiliations:** † Department of Inorganic Chemistry, 16722 (IAdChem) Universidad Autónoma de Madrid, Calle Francisco Tomás y Valiente, 7, 28049 Madrid, Spain; ‡ Department of Molecular and Analytical Chemistry, Interdisciplinary Excellence Centre, 37442 University of Szeged, Dóm tér 7-8, H-6720 Szeged, Hungary; § Department of Medical Microbiology, Albert Szent-Györgyi Health Center and Albert Szent-Györgyi Medical School, University of Szeged, Semmelweis u. 6, H-6725 Szeged, Hungary; ∥ Chair of Medicinal and Bioinorganic Chemistry, School of Natural Sciences, Department of Chemistry, 9184 Technische Universität München, Lichtenbergstrasse 4, 85748 Garching b. München, Germany

## Abstract

The coordination capacity of thiosemicarbazone ligands and their synergism with palladium­(II) ions modulate their reactivity, allowing custom design. Using thiosemicarbazones with two potential stable tautomeric forms and imidazole as bioisosteres, we studied how the substitution in the N4 group of the thiosemicarbazone by the *p*-chlorophenyl group modifies their hydrophilic properties, integrity in solution, and interactions toward their potential targets. The coordination to Pd­(II) affects the bioactivity of the ligands, resulting in either improved or reduced antiproliferative effects depending on the cell type (cancerous versus bacterial, respectively).

## Graphical abstract

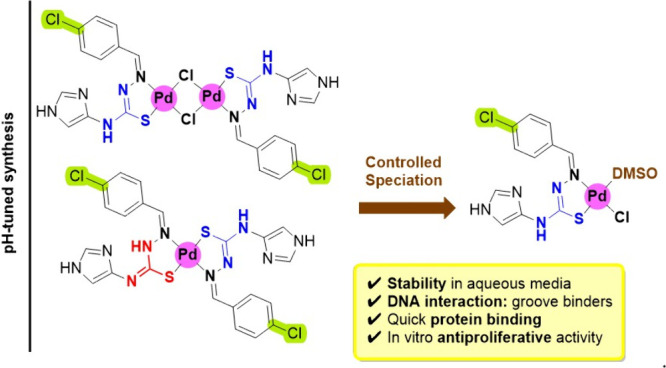

## 1 Introduction

Noncovalent interactions often govern chemical and biological processes. Proteins assemble through the combined effect of various noncovalent interactions (hydrogen bonding, electrostatic interactions, π–π stacking, etc.),^1^ which are critical to understanding their biomolecular structure and function. Therefore, investigating the interactions of metallodrugs, coordinate covalent or noncovalent, with their molecular target is crucial.^2^

Many thiosemicarbazones (TSCs), such as α-heterocyclic, N-substituted derivatives, and those combined with pharmacophoric moieties (i.e., steroids),^3^ are bioactive molecules with a potent capacity to exert antitumor, antibacterial, and antiparasitic action.^4^ Such bioactive moieties are versatile ligands capable of coordinating diverse metal ions. The synergism with metals allows custom designs and has been proven beneficial, contributing to the modulation of their lipophilicity, solubility, reactivity, and interactions with endogenous molecules.^2^ The literature is extensive, and the examples with more than three donor atoms per TSC unit are not limited to only monomers but even include dinuclear complexes or helicates.^3,5,6^ A key discovery is that these TSCs can exist in solution as two equally stable tautomeric forms. We have reported a particularly notable example (Figure 1, ref 7) that exhibits remarkable stability even in biological buffers (containing 1% DMSO). The chemistry of these TSCs, where R_1_ is a N-heterocycle, diverges from that observed in those with a phenyl group (Figure 1, ref 8). On the other hand, the variation in N4-substitution (R_2_ in Figure 1) offers remarkable versatility in applications. For instance, some of these complexes can prevent the precipitation of β-amyloid, while others have demonstrated anticancer activity (Figure 1, R_1_, ref 9), affording a unique, enticing solution speciation that can be controlled using buffers and electrolytes (Figure 1, R_2_, ref 8), in contrast to the rest of the complexes where no transformation processes were detected in solution. The precursor ligands used to isolate the complexes represented in Figure 1 were quite robust in solution, and no transformation processes leading to different species were detected.

**Figure 1:**
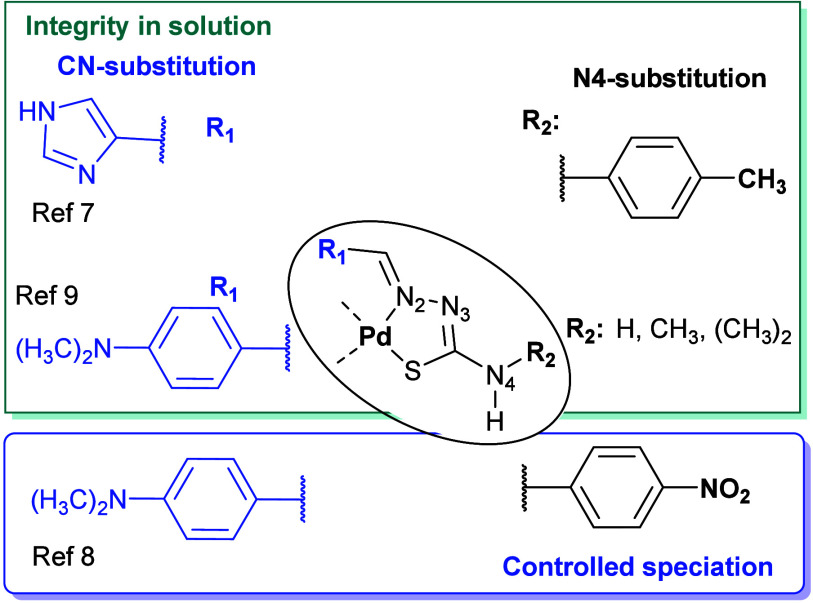
Scheme of our previous work (refs 7–9 ) on the structure–reactivity in solution for active Pd­(II) complexes with a diverse series of TSCs.

Organizing the trends in the antitumor mechanism of action for thiosemicarbazone complexes is challenging due to the variety of metals involved.^10^ Copper­(II) derivatives are the most studied, with some exhibiting dual functionality: inhibiting the R2 subunit of ribonucleotide reductase (RNR) and tubulin polymerization by binding at the colchicine site.^11^ Recently, examples targeting cancer stem cells and reducing pluripotency markers have also been reported,^12^ and some others just targeting mitochondria.^13^ For this work, the literature on Pd­(II) complex mechanisms has been deeply checked. While examples are not numerous, they are quite diverse due to the multiple structures, reactivity, and nuclearity of these metallodrugs. For instance, some Pd complexes are more active than the corresponding Pt­(II) both targeting proteosome.^14^ Research by Jiang *et al.* further illustrates this complexity, showing that cell death results from multiple mechanisms: increased levels of ROS species, DNA damage, and PARP inhibition.^15^ Yang et al. proved Pd­(II) thiosemicarbazones as multitargeting complexes and went a step further using albumin to design a sample preparation protocol at increasing the complex’s specificity and overcoming resistance.^16^

The scientific community is increasingly moving toward standardized protocols in research. This trend aims to generate reliable data and build an extensive knowledge of drug solution profiles. Such a comprehensive understanding will not only boost reproducibility but also shed light on the pharmacokinetic properties and mechanisms of action of drugs. Surprisingly, this crucial information is still missing in recent papers, hindering the comparison of the results of biological activity studies. These studies must begin with a detailed examination of the drug’s solution stability and its interactions with endogenous transporters or target molecules. The nature and strength of these interactions can then be thoroughly evaluated by using thermodynamic studies.

Investigating the lipophilic nature of the potential drug is also an important step, as this physicochemical property strongly influences the aqueous solubility and membrane permeability and has an impact on the cellular uptake and the hydrophobic interactions with biological macromolecules such as proteins or DNA. In the case of metallodrugs, besides binding via secondary interactions where lipophilicity might be a key parameter, coordination of donor atoms of the macromolecules to the metal center can also occur as, for example, in covalent binding metallodrugs. Not only is the knowledge of the solution speciation and lipophilicity important in metallodrug studies, but the choice of the buffer and its impact on the biological content are also crucial.

The interaction of solvents or buffers with a metallodrug is not an obstacle in drug development; in fact, this knowledge will increase the control of this species’ reactivity, facilitate the pharmaceutical preparation, and can also help to overcome drug pollution in water.

In this work, we study the impact of the reactivity of the substitution in the N4 group of the thiosemicarbazone scaffold by the *p*-chlorophenyl group when the imidazole is within the TSC structure. We aim to deepen the knowledge of metallodrugs that produce noncovalent interactions with nontraditional structures, hydrophilic properties, and controlled speciation.

## 2 Results and Discussion

### 2.1 Design and Chemical Synthesis of the Compounds

We reported that α-N-heterocyclic thiosemicarbazones (α-N-TSCs) containing an imidazole moiety can coordinate to the Pd­(II) ion in two distinct tautomers.^7^ We observed that substitutions at the N4 position of the TSC (Figure 1) enhanced its interaction with biological models. Building on these findings, we hypothesize that a *p*-chlorophenyl substitution at this position would exert both electron-withdrawing inductive effects and potentially counterbalance the electron-donating resonance effects on the phenyl moiety.

We synthesized and characterized ligand HL (1*H*-imid­azole-4-carb­oxalde­hyde­(4*N*-*p*-chloro­phen­yl)­thio­semi­carba­zone) and noticed a dramatic change in the reactivity in comparison with our previous results,^7^ which allowed us to achieve three complexes ((1), (2), and (3), see Scheme 1) with structures varying from dinuclear to mononuclear with the general formula [PdL_2_] (with one or two TSC tautomers) or the general formula [PdLCl­(DMSO)] with a single tautomer. We performed the synthesis of the complexes and studied their conversions, varying the concentration, solvent, pH, and temperature. Final conditions are drawn up in Scheme 1 so that we could understand the reactivity of the compounds in solution.

**Scheme 1:**
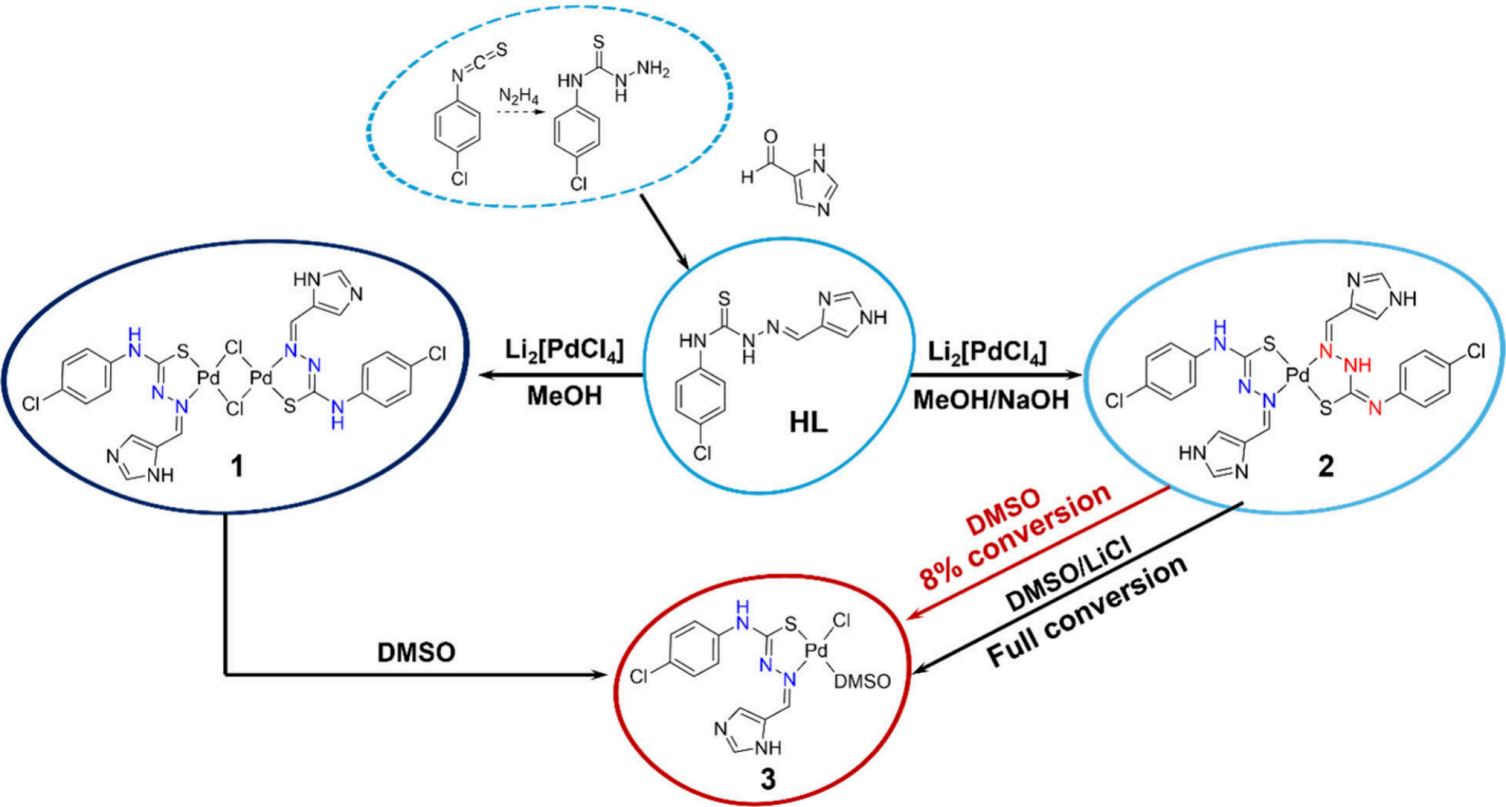
Reactions Performed to Obtain the Compounds Studied in This Work (HL, (1), (2), and (3))

#### 2.1.1 Ligand

##### 2.1.1.1 Design, Synthesis, and Characterization

The ligand (HL) containing a *p*-chlorophenyl substituent was synthesized and characterized, and its protonation processes were studied. The synthesis was performed by a condensation reaction between the 1*H*-imidazole-4-carboxaldehyde and *p*-chloro­phenyl­thio­semi­carb­azide previously prepared from *p*-chloro­phenyl­iso­thio­cyan­ate and hydrazine hydrate. The purity of the ligand was corroborated by elemental analysis and mass spectrometry data. The ^1^H and the ^13^C NMR spectra of the ligand show a higher number of signals than expected for its molecule symmetry (Figure S1.1), which suggests that both tautomeric forms coexist in solution, confirmed by the integration of the peak area. Although this tautomeric equilibrium is like the one detected in previous works,^7^ in this case the thionic form of the ligand becomes predominant at acidic pH values. The change in the predominant tautomeric form is clearly observed when adding D_2_O to the NMR sample in DMSO-*d*_6_, where the pH of the solution changes slightly to 6.80 (Figure S1.2).

##### 2.1.1.2 Ligand p*K*_a_ Studies in Solutions

The protonation state of the compound is a key feature, significantly influencing its average charge, solubility, and lipophilicity. The protonated ligand (H_2_L^+^) has two dissociable protons, namely on the imidazolium nitrogen and the hydrazine nitrogen (NH_hydr_ Figure S2A), and the proton dissociation constants (*K*_a_) were determined in a DMSO/H_2_O solvent mixture (Figure 2) based on the UV–vis spectral changes with varying pH (Figure S2B). Step I starts between pH 3.0 and 6.0 in 60% (v/v) DMSO (red colored spectra), which belongs to the deprotonation of the imidazolium nitrogen (also happening at pH 3.5–7.0 in 30% (v/v) DMSO/H_2_O, spectra not shown).

**Figure 2:**
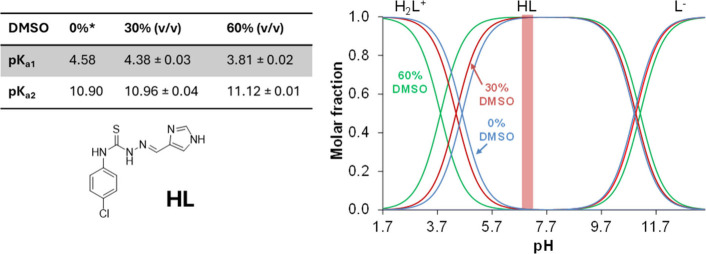
Proton dissociation constants (*K*_a_) of the protonated ligand (H_2_L^+^) and concentration distribution curves, and symbol * is used for predicted values {*c*(HL) = 25.0 μM; *I* = 0.10 M (KCl); 0, 30, and 60% (v/v) DMSO/H_2_O; *t* = 25.0 °C}.

The second deprotonation step is observed in the basic pH range (9.5–12.5 in 30% and 10.0–13.0 in 60% (v/v) DMSO), where extended delocalization of the conjugated electron system in the L^–^ form (Figure S2B,C) leads to a shift in the UV–vis absorption spectrum, with the λ_max_ shifting from 312 to 338 nm in both DMSO/H_2_O solvent mixtures. Figure S2D illustrates the p*K*_a_ values plotted against 1/*ε*_r_ (solvent), where *ε*_r_ is the relative permittivity of the solvent mixture. This plot reveals a positive slope for p*K*_a2_ and a negative slope for p*K*_a1_. This behavior is explained by the Born electrostatic solvent model: as the DMSO content increases, the p*K*_a1_ value (deprotonation of cationic H_2_L^+^) decreases, while the p*K*_a2_ value (deprotonation of neutral HL) increases. Since the poor solubility hampered the determination of the p*K*_a_ values in aqueous solution, the value in pure water is predicted (*value in Figure 2). Based on the p*K*_a_ values, the neutral HL form predominates in a wider pH range including physiologically relevant pH 7.4 (the pH of the extracellular fluids such as blood).

#### 2.1.2 Complex (1)

The reaction of the ligand (HL) with Li_2_[PdCl_4_] at a 2:1 ratio in methanol (MeOH), at pH ∼ 6.5, yields a complex whose elemental analysis and MALDI^+^-MS spectra correspond to the [PdClL]_2_ formula. The ^1^H NMR spectrum of complex (1) exhibits only one set of signals for the phenyl group (single AA′BB′ system) and one set for the imidazolyl substituent. These data suggest that the ligand is coordinated in only one kind of tautomeric form. The structure was further confirmed by ^13^C NMR spectroscopy, in which all signals were identified and assigned (Figure S3). After overnight recording for a better resolution, a new signal was detected in the aliphatic area at 2.54 ppm right beside the DMSO residual peak that corresponds to solvent coordination (Figure S4A). Although this type of dinuclear complex is reported to be quite stable and active on cancer cells,^17^ in our case, the longer periods required for spectra acquisition allowed us to detect the DMSO coordination. The observed cleavage of the Pd–Cl bonds of complex (1) and its poor solubility need to be taken into consideration for further experiments. The difficulty of replicating complex solutions in buffer media that could rapidly provide different species prompted us to discard this complex for biological assays.

#### 2.1.3 Complex (2)

##### 2.1.3.1 Synthesis and Characterization of Complex (2)

Complex (2) was synthesized by adjusting the pH of the HL solution in MeOH to ∼8 using an aqueous 0.1 M NaOH solution. The reaction of this solution with Li_2_[PdCl_4_] affords a new complex whose elemental analysis indicates a [PdL_2_] formula, and the MALDI^+^ mass spectra confirmed a molecular ion at *m*/*z* 664.9 for [M + H]^+^. The ^1^H NMR spectrum at 0.5 mM (Figure S5) shows a set of signals whose number, multiplicity, and integral suggest the coordination of the ligand via its two tautomeric forms. The slightly basic pH used in the synthesis favors the formation of L^–^, producing an immediate coordination to the Pd­(II) center.

The spectrum of complex (2) in DMSO remains stable for up to 24 h, and DMSO coordination is only observed when the concentration exceeds 3 mM (see section 2.1.3.2). The 2D [^1^H, ^13^C] HMQC/HMBC spectra were recorded at 3 mM, and the assignment of the signals is shown in Figure 3.

**Figure 3:**
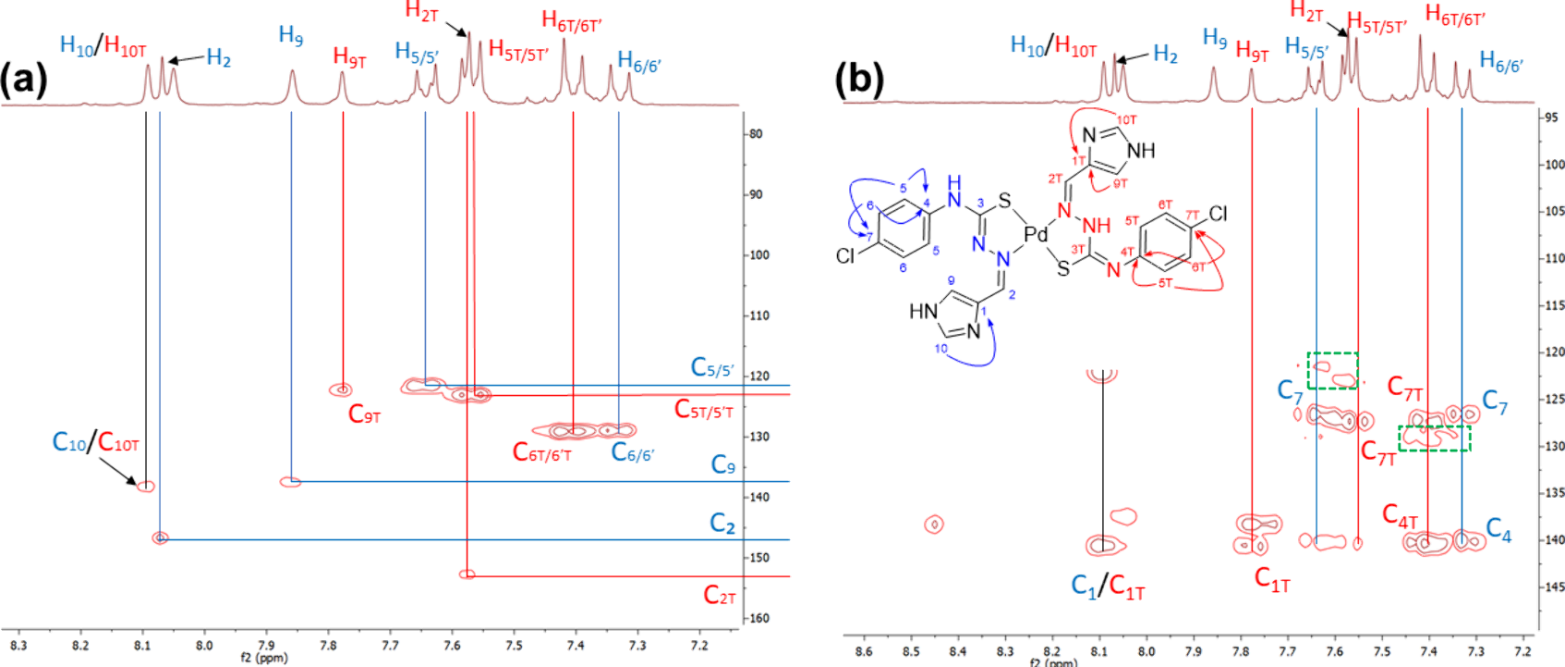
Aromatic region of the 2D (a) HMQC and (b) HMBC spectra of complex (2).

Despite the good match between the elemental analysis data and mass spectra, the 2D spectra at 3 mM showed a minor species at 2.54 ppm (s), and surprisingly, its intensity did not increase over time. The structure of this minor species is suggested based on the two-dimensional spectra (Figure 3), where a small set of signals at 7.31 (d) and 7.63 ppm (d) seems to correspond to a DMSO coordination species (<8%, calculated from the ^1^H NMR spectra). This solution behavior of the coordinated minor species is indicated in Scheme 1 and further clarified and discussed in section 2.1.4.

##### 2.1.3.2 Studies of Complex (2) in Solution

(a) Complex (2) solutions in DMSO (D_2_O) investigated by NMR spectroscopy: Trying to investigate if the higher concentration is responsible for the complex transformation process in DMSO, we monitored the solution of complex (2) at 0.5 and 1 mM by ^1^H NMR spectroscopy (Figure 4a). We could clearly see that complex (2) is only detected pure at a concentration of 0.5 mM. As the concentration of complex (2) increases, the molar fraction of a new coordinated species (complex (3)) gradually rises from 0% (Figure 4a­(3)) at 0.5 mM to 8% at 3 mM (Figure 4a­(1)). Panels (1) and (2) in Figure 4a also show that the signal of complex (3) does not increase over time even after 24 h.

**Figure 4:**
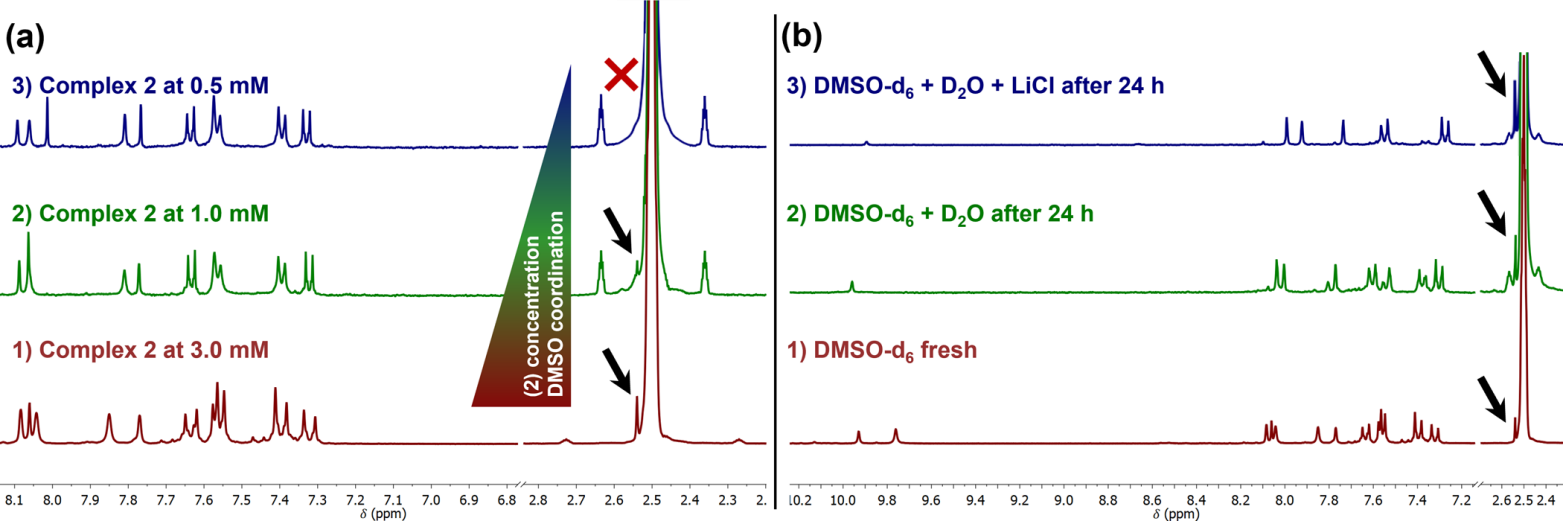
Variation of DMSO coordination of complex (2) (a) with decreasing complex concentration (1–3) and (b) in different solvent mixtures at 3 mM concentration: (1) DMSO-*d*_6_; (2) DMSO-*d*_6_ and D_2_O at *t* = 24 h; and (3) DMSO-*d*_6_ and D_2_O/H_2_O with saturated LiCl at *t* = 24 h.

It has been observed that the addition of LiCl to metal complexes in DMSO solutions (as opposed to NaCl) triggers the coordination of DMSO to the metal center.^8^ To enhance the low level of coordination observed for (2), we added LiCl to its DMSO solution, and the species with completed DMSO coordination (complex (3)) could be achieved even without forcing the conditions (Figure 4b­(3)). This procedure has allowed us to scale and reproduce the reaction to generate complex (3), discussed in the following section (section 2.1.4). The stability of complex (3) was checked in pure DMSO, and no changes were observed over more than 3 days. As coordination of DMSO has been previously reported also in the range of 3.3–3.8 ppm,^18,19^ we have checked this area thoroughly to compare with our different findings (Figures S4B).

(b) Complex (2) p*K*_a_ studies in solution: For these studies, complex (2) was dissolved in 60% (v/v) DMSO/water to replicate the ligand conditions and to prevent this species from precipitating in the whole pH range applied (1.8–14.0). During the spectrophotometric titration of (2), the sample was not protected from light since according to the time-dependent preliminary studies, only minor changes were visible in the first 2.5 h (which is a typical time interval for spectrophotometric titrations). The irradiation study results are reported in Figures S6 and S7.

Spectral changes revealed three equilibrium processes (steps I–III in Figure 5a) without metal complex dissociation. This is supported by an absorption band in the 400–500 nm wavelength range, which is absent in the ligand spectra varying pH. Based on the proton dissociation constants and the spectral changes, we attribute these processes to the deprotonation of the coordinated ligands. All processes are characterized by spectral changes in the 280–480 nm wavelength range, and the three equilibrium processes are easily recognized and separated in Figure S8 by the presence of their corresponding isosbestic points (λ_i.p._ = 300, 398, and 332 nm for steps I, II, and III, respectively, as pH increased, seen in Figure 5a). In the acidic pH range, only one deprotonation process is observed (with p*K*_a1_ = 2.97 ± 0.02), attributable to the deprotonation of an imidazolium nitrogen, as Figure 5a shows. Most probably, the deprotonation of the other imidazolium moiety takes place at strongly acidic conditions (pH < 1.8). This process is followed by the deprotonation of the N_hydrazine_ moieties of the coordinated ligands (p*K*_a2_ = 8.40 ± 0.02, p*K*_a3_ = 11.31 ± 0.01; Figure 5) accompanied by clear spectral changes. The determined proton dissociation constants for steps I and II are reduced by 0.8 and 2.7 logarithm units compared to those of the ligand in the same medium due to coordination to Pd­(II). Based on the concentration distribution curves (Figure 5b), the [PdL_2_H_2_]^2+^ and [PdL_2_H]^+^ species are present at physiologically relevant pH, while the fully deprotonated and neutral complex [PdL_2_] appears at pH > 9.5 and becomes dominant at pH ≥ 11.3.

**Figure 5:**
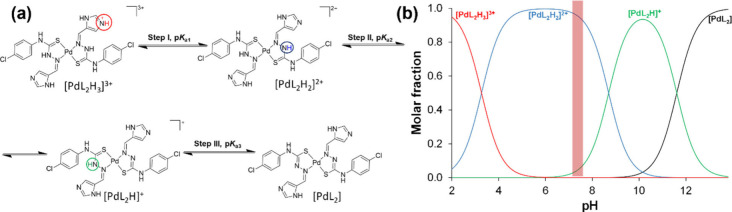
(a) Deprotonation steps of (2), indicating the determined p*K*_a_ values. {solvent: 60% (v/v) DMSO/H_2_O; *I* = 0.10 M (KCl); *t* = 25.0 °C}. (b) Concentration distribution curves for complex (2) species, highlighting the pH range of neutral/physiological conditions. {*c*(complex (2)) = 20.0 μM; *I* = 0.10 M (KCl); 60% (v/v) DMSO/H_2_O; *t* = 25.0 °C}.

#### 2.1.4 Complex (3)

Complex (3) was synthesized by reaction of complex (2) in DMSO with a saturated H_2_O solution of LiCl, following a similar procedure extracted from the literature.^8^ Both the elemental analysis and the mass spectra agree with the proposed molecular formula of [PdLCl­(DMSO)]. The coordination of DMSO appears as a new singlet signal at 2.54 ppm in the ^1^H NMR spectra, which correlates to the corresponding carbon at 40.4 ppm in the 2D [^1^H, ^13^C] HMQC and HMBC spectra (Figure 6). The aliphatic area of both spectra is assigned and inserted between both two-dimensional spectra. In the IR spectrum of complex (3), the most characteristic bands correspond to the ν (S=O) vibration of the coordinated DMSO and the ν (Pd–Cl) stretching band, whose positions are consistent with those reported for analogous complexes in the literature.^20^

**Figure 6:**
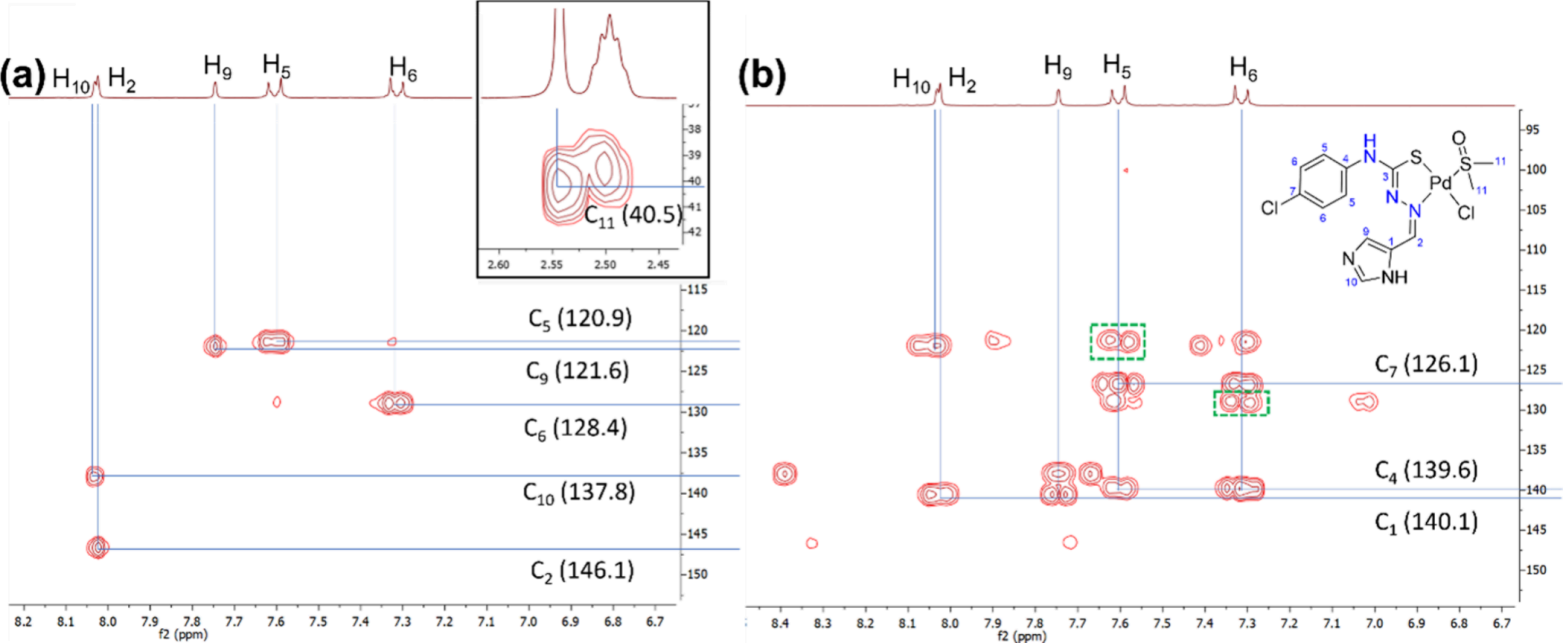
Aromatic region of the 2D (a) HMQC and (b) HMBC spectra of complex (3).

It is worth noting that complex (3) is detected in the DMSO solution of (2) at high concentrations and under visible light (Figures S6 and S7); the levels found in biological milieu are within the micromolar range, and minimum irradiation is contemplated. Complex (2) does not show coordination of DMSO and the chlorido ligand below 0.5 mM, and above that quantity, the presence of (3) does not increase with time, nor does it surpass 8%.

### 2.2 Stability Studies of Complexes (2) and (3)

#### 2.2.1 Stability Studies Using DMSO (1% to 60%) in Physiological Buffers by UV–Vis Spectrophotometry

##### 2.2.1.1 Complexes (2) and (3) in Tris Buffer Solution

Measuring the stability of these metal complexes under simulated physiological conditions (aqueous solution, pH 7.4, *c*(Cl^–^) = 0.1 M, mimicking blood plasma) proved challenging due to their limited aqueous solubility. To address this, various DMSO/H_2_O mixtures were employed as solvents, and the long-term influence of Tris buffer was also investigated. Complex (2) remains stable for up to 24 h in Tris containing 3% DMSO (v/v; Figure S9a), and no precipitation was observed. However, the increase in the DMSO percentage to 60% decreased the stability of complex (2) after 24 h (Figure S9b), and the two characteristic bands of (2) at 343 and 400 nm shifted to lower wavelengths. This observation clearly indicates the occurrence of a transformation process when the concentration is higher. For the sake of comparison, the results of the stability assay are also shown for complex (3) in Figure S9c, where the main absorbance band is seen at 335 nm.

The stability was also assayed at 1% (v/v), and spectra are shown in Figure S10 for comparison. These spectra showed the same profile but with much lower resolution.

##### 2.2.1.2 Complexes (2) and (3) in Phosphate and HEPES Buffers

Measuring the stability of complexes (2) and (3) under the physiological conditions used in section 2.2.1.1, we evaluated the effect of phosphate and HEPES buffers over time at pH 7.4 and 0.1 M chloride concentration. In the presence of phosphate, complexes (2) and (3) precipitated at 3% (v/v) DMSO content, while at 60% (v/v) DMSO, complex (2) remained in solution; however, it suffered from major structural changes, as in its absorption spectra, a new band developed (λ_max_ = 323 nm) during the first 10 h. In HEPES buffer, both complexes formed precipitation at 60% DMSO, also showing spectral changes. Based on these observations, phosphate and HEPES were found to be noninnocent since they reacted with both Pd­(II) complexes, resulting in large spectral changes in the charge transfer bands (see examples in Figure S11). Ligand displacement by coordination of the buffer components is the most plausible reaction.

As mentioned in the previous section, we could use Tris aqueous buffer instead of phosphate or HEPES for monitoring the stability and solubility profile of complex (2). The complex remains stable in solution at 20.0 μM for up to 24 h in Tris containing 3% (v/v) DMSO (Figure S9a) without precipitation. However, increasing the DMSO percentage to 60% significantly decreases the stability of complex (2) (Figure S11), which clearly contrasts with the stability profile observed for phosphate buffer.

### 2.3 HPLC Analysis of the Aqueous Solution Integrity of Complex (2)

HPLC provides precise quantitative results of samples in solutions and ensures the consistent quality of pharmaceutical products, meeting their regulatory requirements, because it delivers repeatable results across different batches. To further assess the stability of complexes (2) and (3), we analyzed their aqueous solutions using general HPLC procedures developed by our research group.^21^

Both complexes were dissolved at a 20 μM concentration in a 3% DMSO aqueous solution and injected as described in the Experimental Section. The chromatograms were collected (Figure 7), showing that the signal from the freshly prepared solutions remained practically unchanged over time. We graphically plotted the stability as the percentage of intact complex remaining with the initial amount (time *t*_0_) set as 100%. The integral of the signal corresponding to the complexes only slightly diminished after 24 h. Importantly, none of the complex signals coincided with that of the ligand, which eluted earlier under the same conditions. Complexes (2) and (3) do not afford any conversion using this concentration under the applied conditions.

**Figure 7:**
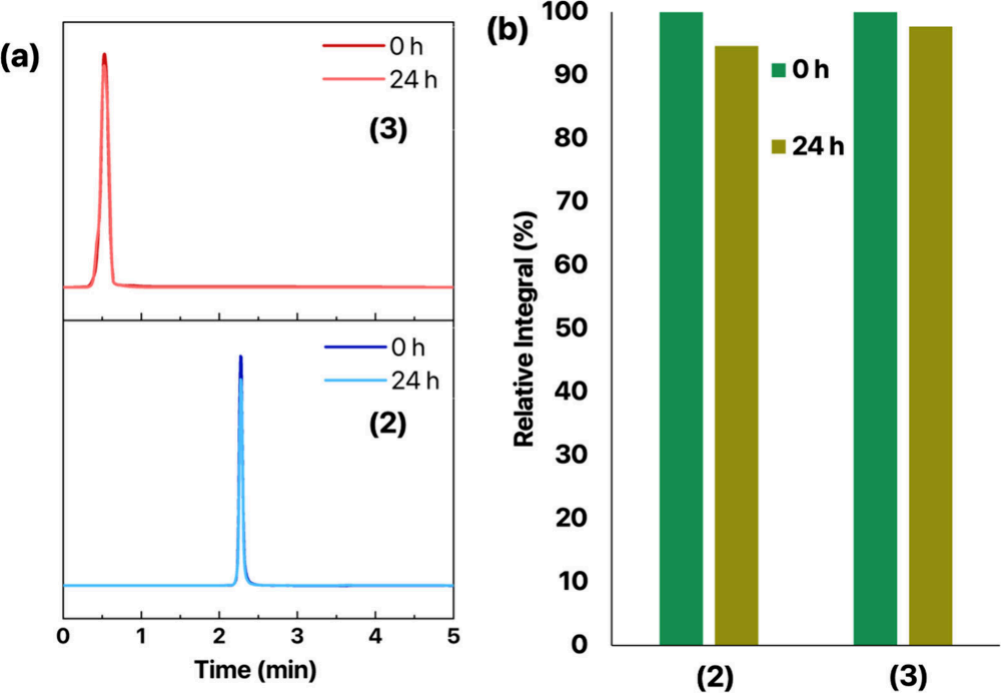
Monitoring the stability of complexes (2) and (3) in aqueous solution (3% DMSO) with HPLC. (a) Chromatograms at 254 nm. (b) Relative integral values.

### 2.4 Lipophilicity of Complexes (2) and (3)

We determined the distribution coefficient (*D*) data through *n*-octanol/H_2_O partitioning in pure water (*D*_o/w_) and in Tris buffer (*D*_o/t_). The presence of buffer ensures physiological pH (7.4) and allows us to maintain the integrity of the diverse models of cellular components and proteins used in this work. Various buffers have been analyzed over the years, and the results indicate that their identity and concentrations (needed to ensure not only the required pH but also the stability of the biological components) need to be carefully tuned for the study of different targets, e.g., diverse proteins. In this work, we have selected Tris buffer following other published procedures.^22^ The use of phosphate and HEPES buffers was not advisable, as the complexes show changes in their UV–visible spectra, indicating structural changes over time (see section 2.2.1.2).

Based on the determined distribution coefficients collected in Table 1, the complexes are more lipophilic than the ligand and may cross the cell membrane easier. The lipophilicity of complex (2) is higher than that of complex (3), and both complexes are more lipophilic in the presence of Tris buffer than in water.

**Table 1:** Log *D*_o/w_ and Log *D*_o/t_ Values

	(2)	(3)	HL
log *D*_o/w_	+1.70 ± 0.06	+1.11 ± 0.06	+0.90 ± 0.08
log *D*_o/t_	+2.32 ± 0.16	+1.94 ± 0.04	+1.58 ± 0.01

### 2.5 Interaction of Complexes (2) and (3) with Calf Thymus-DNA (CT-DNA) Studied by UV–Vis

The UV–vis spectra of CT-DNA show a very intense absorption band at 260 nm, assigned to π–π* electronic transitions in the heterocyclic rings of the nucleotides. The absorbance value of this band is strongly dependent on the conformational changes that can occur in the macromolecule. The interaction of metal complexes with CT-DNA can then be monitored following the changes of this absorption peak. Hyperchromism of the band at 260 nm is usually observed due to the distortion of the secondary structure of DNA caused by coordinate covalent binding or noncovalent interaction in the grooves of the molecule, whereas hypochromism is seen when the complex intercalates between the DNA base pairs.^23^

Figure 8 shows the UV–vis spectra of CT-DNA incubated with increasing concentrations of complexes (2) and (3). As the spectra of both complexes overlap with that of CT-DNA, solutions of the complexes at the same concentration were used as a reference; thus, the presented spectra were corrected by the spectra of the complexes at the corresponding concentrations. In both cases, the absorption values of DNA–complex samples increase with a higher complex concentration. Considering the structures of these complexes, which have no vacant coordination sites conducive to coordinate covalent binding, the data suggest a noncovalent interaction, likely via groove binding.

**Figure 8:**
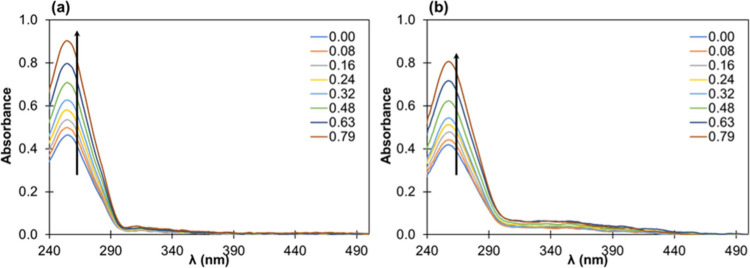
UV–vis spectra of CT-DNA [*c* = 2.54 × 10^–5^ M] incubated with (a) complex (2) (from ratio of 0 to 0.79 as indicated in the figure) and (b) complex (3) (from ratio of 0 to 0.79). 5 mM Tris, 50 mM NaCl, pH 7.40, 25 °C. (The spectra are corrected by the absorption originating from the complex itself.)

### 2.6 Interaction of Complexes (2) and (3) with CT-DNA Studied by Viscometry

To discard other types of interaction, particularly intercalation, we studied the interaction of complexes (2) and (3) with CT-DNA via viscosity measurements. Changes in the DNA chain length produced by metallodrug binding can be distinguished from the different hydrodynamic characteristics coordinate covalent to noncovalent binding modes display.^24^ As such, viscosity (η) is a direct parameter for studying drug–DNA interactions as a function of the hydrodynamic changes induced by a binding agent. The relative viscosity (η/η_0_) is directly proportional to the cube of the contour length (*L*) of DNA: η/η_0_ = (*L*/*L*_0_)^3^. For example, the presence of an intercalating molecule (such as ethidium bromide) increases the dynamic viscosity of the solution containing the DNA because the DNA double helix is lengthened (Figure 9, yellow squares). Groove binders such as Hoechst 33258 fluorescent dye do not alter the length of the DNA significantly, causing minimal changes in the viscosity of the solution. In the case of a coordinate covalent binder such as cisplatin, the relative viscosity of the solution decreases (Figure 9, green diamonds).^24^ Figure 9 shows the variation in the viscosity of CT-DNA solution upon the addition of the complexes (2) and (3).

**Figure 9:**
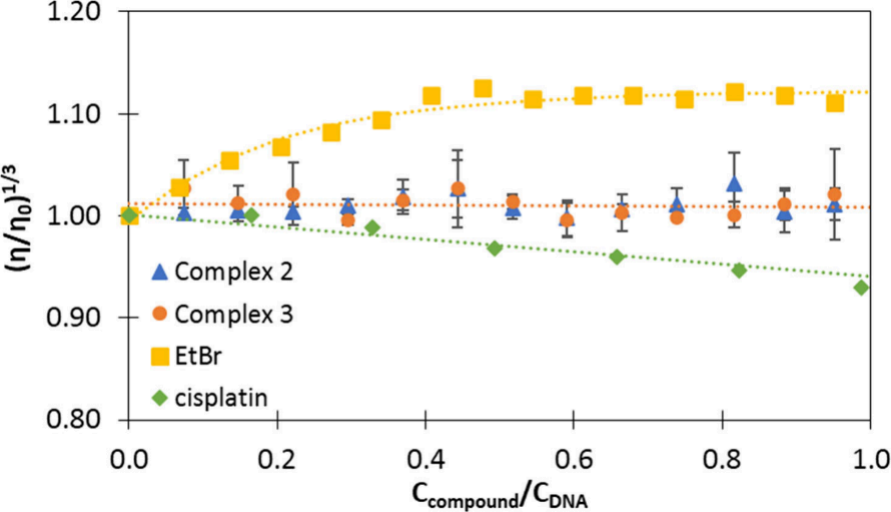
Effect of increasing amounts of complexes (2) and (3) on the viscosity of CT-DNA. Cisplatin and ethidium bromide (EtBr) are included as references (diamonds and squares, respectively). The data were collected for *c*_DNA_ = 5.69 × 10^–5^ M and different *c*_compound_/*c*_DNA_ ratios from 0.0 to 1.0, in triplicate). 5 mM Tris, 100 mM NaCl, pH 7.40, 25.0 ± 0.1 °C.

As shown in Figure 9, the addition of the palladium complexes does not significantly change the relative viscosity of CT-DNA. This viscosity profile is more in agreement with that of typical DNA groove binders instead of intercalators or covalent binders; thus, groove binding is suggested as the most plausible mechanism of interaction.^8,25^

### 2.7 Interaction of Complexes (2) and (3) with pBR322 Studied by Electrophoresis

To prove our hypothesis, we performed gel electrophoresis assay with a bacterial DNA plasmid (pBR322), as it is a versatile DNA model for evaluating the interaction of the platinum compounds.

Electrophoretic mobility shift assays can be employed to determine the influence of drug interactions on DNA supercoiling. The assay with two forms (relax mode) helps to reveal covalent binders like cisplatin.^24^ The electrophoresis of the plasmid pBR322 (two forms) in the presence of complexes (2) and (3) and cisplatin is shown in Figure S12. As reported, cisplatin is bound via a coordination bond and produces from lanes 11 (its lower concentration) to 14 changes in the mobility of the pBR322 isoforms, slowing the closed circular supercoiled (SC) form (unwinding produced by covalent interaction) and increasing the mobility of the open circular (OC) form (“platination reaction”) until both comigrate. None of our complexes seem to produce a coordination bond (lanes 3 to 10, Figure S12).

The electrophoretic mobility of the supercoiled plasmid pBR322 (pure form) with (2) and (3) evidences the unwinding effect produced by noncovalent interactions (Figure 10). A small unwinding gives rise to a small OC isoform band, observed in lanes 7 to 10 for (2) and lanes 11 to 14 for (3) (Figure 10). This small unwinding does not increase with concentration and does not show the distinct “up and down” migration pattern evident of an intercalating agent,^8,26^ which further underpins a groove binding mode of interaction of the complexes with DNA.

**Figure 10:**
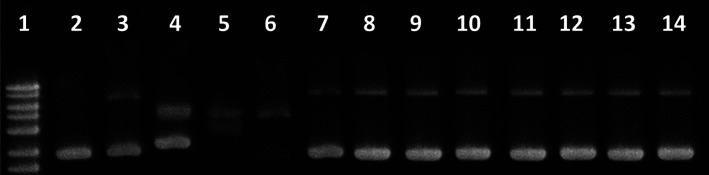
Gel electrophoresis. Lane 1: 1 kb DNA ladder; lane 2: pBR322 control; lanes 3–6: cisplatin at *r*_i_: 0.01, 0.05, 0.10 and 0.20; lanes 7–10: complex (2) at *r*_i_: 0.01, 0.05, 0.10 and 0.20; and lanes 11 to 14: complex (3) at *r*_i_: 0.01, 0.05, 0.10, and 0.20. *c*_DNA_ = 0.0625 μg/μL.

### 2.8 Interaction with DNA G-Quadruplexes (G4s) Studied by FRET DNA Melting

To assess the binding affinity of the compound toward other noncanonical secondary DNA structures, the stabilization of DNA guanine quadruplexes (G4s) by the Pd­(II) compounds was evaluated by FRET (Förster resonance energy transfer) DNA melting assay according to previously established procedures.^27^ G4s appear in guanine-rich sequences, formed by stacks of guanosine quartets; each quartet is assembled via Hoogsteen base pairing in a planar arrangement, and the secondary stacking of the tetrads is stabilized by K^+^ ions.^28^ G4 structures are found in telomeres and promoter regions of oncogenes, and their stabilization by small-molecule compounds, including metal complexes,^29–32^ results in the inhibition of telomerase activity and is associated with anticancer activity. Thus, two representative G4 models were studied, namely a telomeric sequence (hTel21: 5′-GGG­(TTA­GGG)_3_-3′) and a promoter one (C-KIT1: 5′-AGGG­AGGGCG­CTGGG­AGGA­GGG-3′). The obtained results (Figure 11) reveal only moderate G4 stabilizing effects of the compounds; although the changes in the medium melting temperature (Δ*T*_m_) are modest (Figure 11c), the Pd­(II) complexes stabilized more than the free ligand (HL), particularly in the case of hTel21.

**Figure 11:**
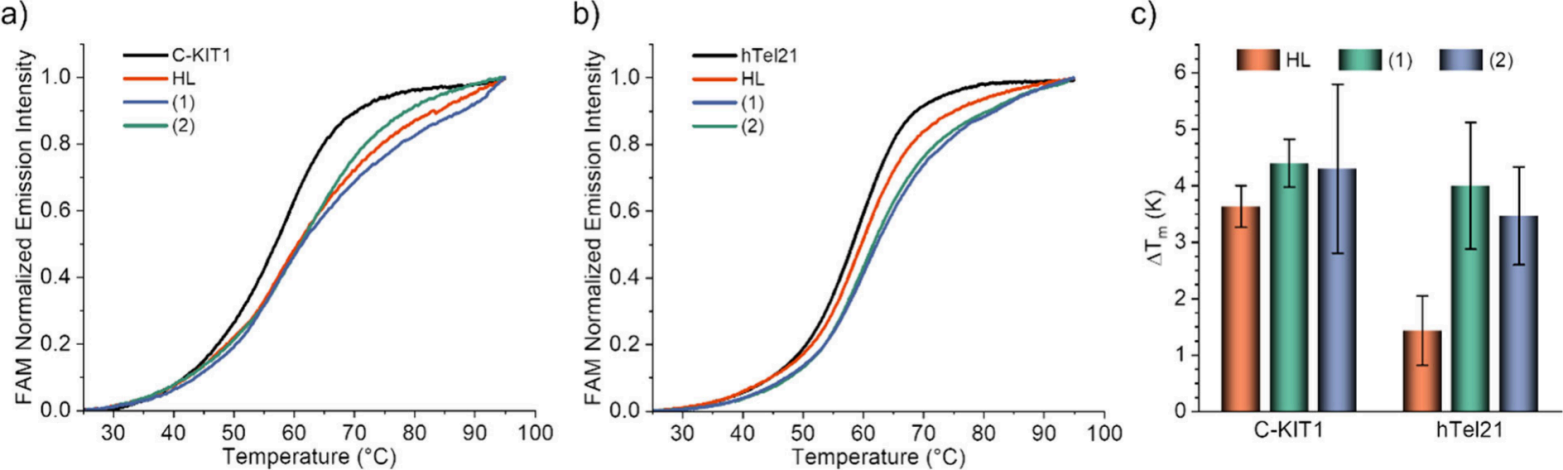
FRET-determined DNA melting curves of (a) C-KIT1 and (b) hTel21 showing the observed changes in the presence of 5 equiv of the different compounds. (c) Δ*T*_m_ induced by the different compounds in C-KIT1 and hTel21.

### 2.9 Interaction of Complexes (2) and (3) with Model Proteins Studied by UV–Vis

#### 2.9.1 Reaction with Glutathione

The interaction of Pd­(II) metal complexes with the tripeptide glutathione (γ-l-Glu–l-Cys–Gly; GSH) is worth studying due to the detoxification role of this abundant intracellular thiol.^33,34^ GSH has a role in the removal of transition metal ions and their complexes, including some platinum anticancer compounds.^35^ Resistance to the widely used cisplatin is GSH-dependent, which results in difficulties in the therapy. Complex [Pt­(GS)_2_] is formed from cisplatin as it was first observed in leukemia cells.^33^ The transmembrane pump ABCC2 exports [Pt­(GS)_2_], while ABCC1 and ABCC2 can transport various metal–GSH adducts including glutathione complexes of copper, mercury, and arsenic.^33,36^

The reactions between Pd­(II) complexes (2) and (3) using micromolar concentrations and GSH were studied under physiologically relevant conditions in the presence of a large excess (30-fold) of GSH. Both (2) and (3) showed relatively rapid interaction with GSH, as the reaction reached the equilibrium state within 30 min (Figure 12). The liberation of at least one thiosemicarbazone ligand and formation of the [Pd­(L)­(GS)] ternary adduct are assumed in the first step. In other examples of palladium complexes, the formation of even higher oligonuclear complexes has been observed with the participation of one or two GSH molecules, similarly as it was found with Cys, confirmed by X-ray crystallography.^37,38^ Based on these results and the similarities observed in Figure 12, complexes (2) and (3) might dissociate intracellularly where GSH has a millimolar concentration.

**Figure 12:**
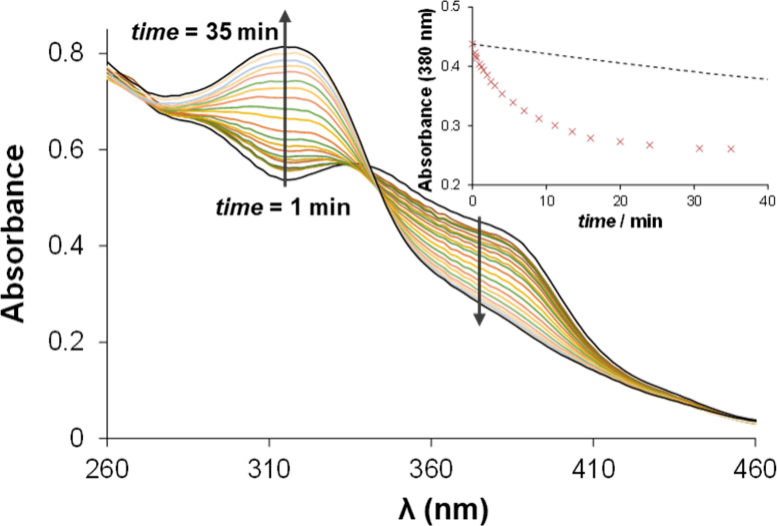
Time-dependent UV–vis absorption spectra of complex (2) in the presence of 30-fold GSH. Inset shows the change of absorbance at 380 nm (×) compared to the change in the presence of phosphate under the same conditions (dashed line). {*c*(2) = 20.0 μM; *c*(GSH) = 600 μM; *I* = 0.10 M (KCl); solvent: 60% (v/v) DMSO/H_2_O; *t* = 25.0 °C}.

#### 2.9.2 Interaction with Lysozyme

Even though DNA is probably the main target for this complex, the interaction of potential metallodrugs with proteins is often an additional key in the mechanism. For this reason, the interaction of complexes (2) and (3) with a model protein, namely HEWL (hen egg white lysozyme), was also studied. This protein was selected because it is broadly employed to study the interaction of proteins with metallodrugs. Moreover, the significant role of disulfide bonds in their stability has been proven.^36,39^

Lysozyme has a significant absorption band at 280 nm due to π–π* and n−π* transitions due to the aromatic amino acids tyrosine and tryptophan and phenylalanine to a lesser extent. The observed rate constant (*k*_obs_) for the reaction of the complex with the protein was calculated monitoring this absorption band at 280 nm as a function of time as pseudo-first-order kinetics, as explained in the experimental part (Figure S13). The calculated values for complexes (2) and (3) are *k*_obs_ = (8.83 ± 0.05) × 10^–5^ and (8.75 ± 0.09) × 10^–5^ s^–1^, respectively. Both complexes thus have a lower reaction rate to lysozyme in comparison to cisplatin (*k*_obs_ of 1.98 × 10^–4^ s^–1^ reported under the same conditions).^9^

### 2.10 *In Vitro* Cytotoxicity in Cancer Cell Lines and Antibacterial Effect

Thiosemicarbazones and their metal complexes often have cytotoxic effects on various cancer cell lines. Imidazole-derived thiosemicarbazones showed no or a moderate cytotoxic effect, while their mixed-ligand Cu­(II) complexes with dichloroacetate had improved cytotoxicity in the same cells.^40^ There are examples for Pd­(II) complexes that displayed considerably higher antiproliferative activity than cisplatin.^41,42^ Herein, the *in vitro* cytotoxicity of HL and complexes (2) and (3) was assayed, and the results are collected in Table 2, expressed as IC_50_ values (using a 72 h incubation period). Complex (2) exhibited the highest cytotoxic activity, whereas HL had half the activity in both cancer cell lines. However, the partial replacement of the TSC ligand in the complex by GSH, which is found in high concentration in the cytosol, especially in cancer cells, cannot be excluded. The release of the TSC ligand from the complex can contribute to the obtained lower IC_50_ value. Complex (3) had the lowest anticancer activity in MCF7 cancer cells. The complexes produce lower values than other metallodrugs such as cisplatin,^43,44^ used in clinic. Doxorubicin, an anthracycline used in neoadjuvant chemotherapy with cisplatin, is also included for comparative purposes.

**Table 2:** *In Vitro* Cytotoxicity (IC_50_ Values Expressed in μM in Colo 205 Human Colon Cancer and MCF7 Human Breast Cancer Cell Lines) of the Title Compounds (72 h Exposure)

	Colo 205	MCF7
HL	19.14 ± 0.60	2.97 ± 0.32
(2)	11.77 ± 0.35	1.55 ± 0.03
(3)	16.24 ± 1.18	8.44 ± 1.13
Cisplatin	23 ± 1	15 ± 0.01
Doxorubicin	0.158 ± 0.004	0.128 ± 0.0009

Since cancer and its medication with chemotherapeutic drugs weaken the immune system, bacterial infections can be life-threatening during treatment. The antibacterial effects of these compounds were also tested on different bacterial strains (Table S1). It was found that only the ligand HL exhibited a moderate-to-low antibacterial effect, which disappeared after complexation with Pd­(II).

## 3 Conclusions

1*H*-Imid­azole-4-carb­oxalde­hyde­(4*N*-*p*-chloro­phen­yl)­thio­semi­carba­zone reacts with palladium­(II), giving three different complexes: a binuclear complex with chlorido bridges, a mononuclear complex in which the ligand is coordinated in two different tautomeric forms, and a species in which the solvent DMSO is coordinated. We studied their transformation, varying the concentration, solvent, pH, and temperature, establishing the conditions for the integrity of every species in solution. Introducing an electron-withdrawing *p*-chlorophenyl group at the N4 position of the ligand generated a new series of compounds. This series exhibits complexes (bearing equal or different ligand tautomers) and a reactivity profile that can be controlled by manipulating the pH and concentration in the presence of DMSO. The anticancer activity of these Pd­(II) complexes is higher than that of the ligand; however, partial release of the ligand from the complexes upon reaction with GSH cannot be excluded, leading to stronger activity. The coordination to palladium decreased the antibacterial activity of the ligand. Their interaction toward biological targets is quite different to cisplatin, being groove binders toward DNA and weaker binders toward lysozyme. These differences, together with the studies indicating how to preserve their stability in solution, prove them to be good candidates for further studies toward experimental therapeutics.

## 4 Experimental Section

### 4.1 Materials and Methods

#### 4.1.1 Chemicals and Biological Stock Solutions

The chemicals were purchased from Johnson Matthey, Sigma-Aldrich, and VWR. pBR322 plasmid was purchased from Fisher Scientific, and calf thymus-DNA (CT-DNA), as lyophilized sodium salt, was purchased from Sigma-Aldrich (ref number D1501, CAS 73049-39-5). The stock solutions were prepared by dissolving known amounts in 5 mM Tris/50 mM NaCl buffer and were standardized spectrophotometrically (ε = 6600 M^–1^ cm^–1^ at 260 nm). Ethidium bromide was purchased from Sigma. Its stock solution was prepared by dissolving suitable amounts of the solid in water and was standardized spectrophotometrically (ε = 5700 M^–1^ cm^–1^ at 480 nm).

#### 4.1.2 Methods

NMR spectra were recorded at room temperature, using a two-channel 300 MHz Bruker Avance III-HD Nanobay spectrometer equipped with a 5 mm BBO 1H/X probe and Z gradients, located at the Interdepartmental Investigation Service (SIdI). DMSO-*d*_6_ was used as the solvent (containing 0.05% (v/v) tetramethylsilane (TMS) as a reference). Chemical shift values are given in parts per million relative to the residual TMS signals. The following abbreviations were used: s (singlet), d (doublet), and m (multiplet). Elemental analyses were performed on a LECO CHNS-932 elemental analyzer located at SIdI. Mass spectra were recorded using FAB (fast atom bombardment) in a Waters VG AutoSpec mass spectrometry unit and using MALDI (matrix-assisted laser desorption/ionization) with a Bruker Ultraflex III (MALDI-TOF/TOF) mass spectrometry unit, both located at SIdI. Infrared (IR) spectra were recorded using a PerkinElmer Model 283 spectrometer with an attenuated total reflectance (ATR) MIRacle Single Reflection Horizontal accessory and equipped with CsI optical windows for spectra between 600 and 200 cm^–1^ in Nujol mull preparations. Absorbance spectra were recorded using a Thermo Evolution 220 spectrophotometer to record the UV–visible region, equipped with temperature control within ±0.1 °C. Viscosity measurements were performed using an A&D SV-1A vibro viscometer. HPLC analyses were carried out utilizing a 1200 Infinity series HPLC system provided by Agilent Technologies coupled to a photodiode array detector.

### 4.2 Chemical Synthesis

#### 4.2.1 Ligand

The ligand synthesis required *p*-chloro­phenyl­thio­semi­carba­zide as a starting material, which was prepared following a reported procedure, with small variations.^45^ Briefly, 20 mL of an ethanolic solution of hydrazine hydrate (0.500 g; 10 mmol) was added over an ethanolic suspension of *p-*chloro­phenyl­iso­thio­cyan­ate (0.848 g; 5 mmol) cooled in an ice bath under constant stirring for 1 h. The white precipitate that formed was then filtered, washed with cold ethanol and diethyl ether, and finally vacuum-dried.

*p*-Chloro­phenyl­thio­semi­carba­zide: White solid. Yield: 77%. Elemental analysis (%): experimental: C, 41.47; H, 3.88; N, 21.09; calcd for C_7_H_8_ClN_3_S: C, 41.69; H, 4.00; N, 20.84. ^1^H NMR [DMSO-*d*_6_], δ (ppm): 9.23 (s, 1H, *N2H); 7.69 (d, 2H, H4); 7.34 (d, 2H, H3); 4.87 (s, 2H, *N1H). IR (KBr, cm^–1^): ν_a_ (NH_2_): 3293; ν (NH): 3177; ν (CH sp^2^): 2940; δ (NH_2_): 1635; thioamide I (C=N): 1533; thioamide IV (C=S): 906.

The ligand (HL) was then synthesized as follows: A solution of *p*-chloro­phenyl­thio­semi­carba­zide (0.403 g; 2 mmol) in 20 mL of ethanol and 5 mL of 6% aqueous acetic acid was added dropwise to an ethanolic solution (10 mL) of 1*H*-imid­azole-4-carb­oxalde­hyde (0.192 g; 2 mmol). The reaction mixture was kept under constant stirring and at reflux temperature for 5 h. The final yellowish solution was then concentrated until a white solid precipitated, which was then filtered, washed with a hot 6% acetic acid aqueous solution, cold water and diethyl ether, and finally vacuum-dried.

1*H*-Imid­azole-4-carb­oxalde­hyde­(4*N*-*p*-chloro­phen­yl)­thio­semi­carba­zone (HL): White solid. Yield: 76%. Elemental analysis (%): experimental: C, 46.97; H, 3.71; N, 24.49; calcd for C_11_H_10_ClN_5_S: C, 47.23; H, 3.60; N, 25.04. ^1^H NMR [DMSO-*d*_6_], δ­(ppm): 13.48 (s, 1H, *N1H); 8.05 (s, 1H, H2); 7.84 (s, 1H, *N2H); 7.74 (s, 1H, H9); 7.66 (d, 2H, H5); 7.41 (s, 1H, *N3H); 7.37 (d, 2H, H6); 7.37 (s, 1H, H10). ^13^C NMR [DMSO-*d*_6_], δ­(ppm): 175.6 (C3), 175.3 (C3T), 138.1 (C7), 137.9 (C7T), 136.8 (C2), 135.4 (C1), 131.5 (C10), 129.2 (C4), 128.9 (C4T), 128.1 (C6), 127.9 (C6T), 126.8 (C5), 126.4 (C5T), 122.1 (C9). IR (cm^–1^): ν (NH imidazole): 3323; ν (NH): 3194; ν (NH): 3121; ν (CH sp^2^): 3012; ν (C=N): 1613; thioamide I (C=N): 1529; thioamide IV (C=S): 831. ESI^+^-MS (*m*/*z*): [M + H]^+^ = 280.

#### 4.2.2 Palladium Complexes

##### 4.2.2.1 Complexes (1) and (2)

First, Li_2_[PdCl_4_] was generated *in situ* following the reported procedure, from PdCl_2_ (0.048 g; 0.25 mmol) and LiCl (0.043 g; 1 mmol) in 10 mL of methanol and under an argon atmosphere. A solution of the ligand HL (0.100 g; 0.50 mmol) in 15 mL of methanol was added dropwise over Li_2_[PdCl_4_] for (2), and for (1), this solution was previously basified to pH 8 using aqueous 0.1 M NaOH. In both cases, the reaction mixture was kept at 40 °C and under constant stirring for 4 h. A yellow precipitate appeared, which was filtered, washed with distilled water and hot methanol, and finally vacuum-dried.

Di-μ-chlorido-bis­(1*H*-imida­zole-4-carb­oxalde­hyde-4*N*-*p*-chloro­phenyl­thio­semi­carba­zonato)­di­palla­dium­(II), complex (1): Yellow-orange solid. Yield: 69%. Elemental analysis (%): experimental: C, 31.21; H, 2.73; N, 16.34; calcd for C_22_H_18_Cl_4_N_10_Pd_2_S_2_: C, 31.41; H, 2.16; N, 16.65. ^1^H NMR [DMSO-*d*_6_], δ­(ppm): 13.39 (s, 2H, *NH); 9.94 (s, 2H, *NH); 8.09 (s, 2H, H10); 8.07 (s, 2H, H2); 7.78 (s, 2H, H9); 7.65 (d, 4H, H5); 7.33 (d, 4H, H6). ^13^C NMR [DMSO-*d*_6_], δ­(ppm): 146.1 (C2), 140.1 (C1), 139.6 (C4), 137.8 (C10), 128.4 (C6), 126.1 (C7), 121.6 (C9), 120.9 (C5), 173.5 (C3). MALDI^+^-MS (*m*/*z*): [M – Cl]^+^ = 805. IR (ATR, cm^–1^): ν (NH): 3314; ν (C=N): 1592; thioamide I (C=N): 1521; thioamide IV (C–S): 817.

Bis­(1*H*-imida­zole-4-carbox­alde­hyde-4*N*-*p*-chloro­phenyl­thio­semi­carba­zon­ato)­palla­dium­(II), complex (2): Yellow-orange solid. Yield: 74%. Elemental analysis (%): experimental: C, 39.56; H, 3.10; N, 20.82; calcd for C_22_H_20_Cl_2_N_10_PdS_2_: C, 39.68; H, 3.03; N, 21.03. ^1^H NMR [DMSO-*d*_6_], δ­(ppm): 13.38 (s, 1H, *NH); 12.86 (s, 1H, *NH); 12.07 (s, 1H, *NH); 10.30 (s, 1H, *NH); 10.13 (s, 1H, *NH); 9.93 (s, 1H, *NH); 9.76 (s, 1H, *NH); 8.08 (s, 1H, H10/H10T); 8.06 (s, 1H, H2T); 8.04 (s, 1H, *NH); 7.85 (s, 1H, H9T); 7.60 (m, 4H, H5/H5T); 7.37 (m, 4H, H6/H6T); 7.77 (s, 1H, H9); 7.64 (d, 2H, H5T); 7.57 (d, 2H, H5); 7.56 (s, 1H, H2); 7.40 (d, 2H, H6); 7.33 (d, 2H, H6T). HMQC, HMBC [^1^H, ^13^C] [DMSO-*d*_6_], δ­(ppm): 152.6 (C2), 146.6 (C2T), 140.5 (C1), 140.5 (C1T), 140.3 (C4), 140.1 (C4T), 138.2 (C10T), 138.1 (C10), 137.5 (C9), 129.1 (C6T), 128.8 (C6), 127.3 (C7), 126.4 (C7T), 123.0 (C5), 122.2 (C9T), 121.4 (C5T). ESI^+^-MS (*m*/*z*): [M + H]^+^ = 665. IR (ATR, cm^–1^): ν (NH): 3331; ν (C=N): 1597; thioamide I (C=N): 1536; thioamide IV (C–S): 821; ν (Pd–N): 362; ν (Pd–S): 353.

##### 4.2.2.2 Complex (3)

(3) was obtained by an already reported procedure, with slight variations.^8^ Briefly, complex (2) (0.02 g; 0.03 mmol) was dissolved in 10 mL of DMSO. To this solution, 10 equiv of LiCl dissolved in water (5 mL) was added, and the mixture was left stirring at room temperature for 24 h. The final complex (3) was then precipitated with ice-cold distilled water (20 mL), washed with water and hot methanol, and finally vacuum-dried.

Chlorido­(dimethyl sulfoxide)­(1*H*-imida­zole-4-carbox­aldehyde-4*N*-*p*-chloro­phenyl­thio­semi­carba­zon­ato)­palla­dium­(II), complex (3): Brown solid. Yield: 83%. Elemental analysis (%): experimental: C, 30.98; H, 3.27; N, 13.83; calcd for C_13_H_15_Cl_2_N_5_OPdS_2_: C, 31.31; H, 3.03; N, 14.04. ^1^H NMR [DMSO-*d*_6_], δ (ppm): 2.54 (s, 6H, H11); 7.31 (d, 2H, H6); 7.60 (d, 2H, H5); 7.74 (s, 1H, H9); 8.02 (s, 1H, H2); 8.03 (s, 1H, H10); 9.91 (s, 1H, *NH); 13.32 (s, 1H, *NH). HMQC, HMBC [^1^H, ^13^C] [DMSO-*d*_6_], δ­(ppm): 146.1 (C2), 140.1 (C1), 139.6 (C4), 137.8 (C10), 128.4 (C6), 126.1 (C7), 121.6 (C9), 120.9 (C5). MALDI^+^-MS (*m*/*z*): [M – Cl]^+^ = 460. IR (ATR, cm^–1^): ν (NH): 3249; ν (C=N): 1597; thioamide I (C=N): 1549; ν (S=O): 1085; thioamide IV (C–S): 819; ν (Pd–N): 368; ν (Pd–S): 353; ν (Pd–Cl): 303.

### 4.3 Stability Studies

#### 4.3.1 HPLC Methods and Sample Preparation

Ligand, complex (2), and complex (3) solutions were prepared fresh as described in the UV–vis stability assay. Prior to the injection to the column, 20 μL was taken and diluted with 180 μL of HPLC-grade acetonitrile at various time points (*t* = 0 and 24 h). 20 μL of this diluted sample was injected in the Zorbax Eclipse Plus C18 column (4.6 × 100 mm, 3.5 μm): flow rate, 1 mL/min; detection, UV 254 nm; flow solvent system A/B (acetonitrile/water, v/v): 70:30 and analyzed with 254 nm radiation up to 10 min.

#### 4.3.2 Stability Monitored by UV–Visible Spectroscopy

The stability of the stock solutions and the effect of diffuse solar irradiation were monitored in DMSO by UV–visible (UV–vis, see Methods) spectra in the 260 to 860 nm wavelength range, and the path length was 1 cm. The initial sample was divided equally into two portions for the stability studies; one portion was irradiated with diffuse light and followed over time, while the other one was stored, protected from light, and measured 1 day after dissolution.

The reaction of the metal complexes (2) and (3) with buffer components was followed in an array of DMSO percentages, from 1% to 60% (v/v) DMSO/H_2_O (pH 7.4) medium. The samples were prepared by dilution of the stock solution of the respective complex in DMSO (5 mM) with 5 mM Tris 1% (v/v) DMSO/H_2_O or 5 mM phosphate buffers 60% (v/v) DMSO/H_2_O (pH 7.40 in both cases), reaching a final concentration of 10^–6^ to 10^–4^ M. The UV–vis spectra of the final samples were followed over time at 37 °C for up to 24 h.

#### 4.3.3 Spectrophotometric Titrations

Spectrophotometric titrations were applied to study the equilibrium processes (proton dissociation and complex dissociation/formation) in DMSO/H_2_O mixtures due to the limited aqueous solubility of the compounds. The calibration of the electrode system was performed by strong acid–strong base pH-potentiometric titrations in 30% (v/v) and 60% (v/v) DMSO/H_2_O solvent mixtures at 25.0 ± 0.1 °C as described in our previous works.^46,47^ KCl (0.1 M) was the supporting electrolyte to maintain constant ionic strength, which refers to the chloride ion concentration in blood plasma. Proton dissociation constants (*K*_a_) of the fully protonated ligand (H_2_L^+^) and its complexes were determined using low concentrations (20–25 μM) of the compounds. The initial sample volume was 5 mL. Spectrophotometric titrations were performed in the pH range 1.8–12.5 for 30% (v/v) DMSO/H_2_O medium and 1.8–14.0 for 60% (v/v) DMSO/H_2_O medium. Prior to the titrations, purified argon was passed through the samples for approximately 10 min to remove CO_2_ traces, and argon was also passed over the samples during these measurements. The proton dissociation constants were calculated by using the computer program PSEQUAD.^48^

### 4.4 Lipophilicity Measurements

The distribution coefficient values (*D*_7.4_) of the compounds were determined by the traditional shake-flask method in *n*-octanol/buffered aqueous solution at pH 7.40 for 5 mM Tris HCl with 0.1 M KCl and at pH 7.4 at 25.0 ± 0.2 °C. The ligand and complexes were dissolved in *n*-octanol (presaturated by the aqueous buffer) because of the very low aqueous solubility. The aqueous buffer solution and *n*-octanol ratio was 1:1 due to the expected high lipophilicity of the compounds. First, samples were mixed with vertical rotation (∼20 rpm) for 3 h, and then the two phases were separated by centrifugation at 3000 rpm (∼1000*g*) for 3 min. After separation, UV–vis spectra of the compounds were recorded in both phases. Spectra of the *n*-octanol phases were compared to those of the original *n*-octanol stock solutions.

The distribution coefficient values were also determined using unbuffered water and octanol (also presaturated) with a ratio of 1:1.

### 4.5 DNA Interaction Studies

#### 4.5.1 UV–Vis Titrations

To investigate the potential binding ability of the palladium complexes (2) and (3) with DNA, UV–vis titrations (240 to 700 nm) were performed at room temperature. During the titrations, the CT-DNA concentration (5.08 × 10^–5^ M) was kept constant, while the concentration of each complex (0 to 2.00 × 10^–5^ M) was varied. The changes in the typical absorbance of CT-DNA at 260 nm were monitored after equilibration (10 min) and after 1 h of incubation at 37 °C. The studies were performed in 5 mM Tris buffer containing a maximum of 3% (v/v) DMSO in the final solution, a concentration in which the stability has been proved and correlated with the 3% DMSO profile. Control experiments with DMSO were performed, and no changes in the CT-DNA spectrum were observed.

#### 4.5.2 Viscosity Assays

The sample preparation for viscometry assay was carried out using the same protocol indicated in section 4.5.1 but only with 5 mM Tris 1% (v/v) DMSO/H_2_O. Then, the samples were incubated at 37 °C and with constant shaking at 300 rpm for 10 min and 1 h. After each incubation, the viscosity was measured at a constant temperature of 25.0 ± 0.2 °C in a vibrational viscometer. The relative viscosity was calculated by taking the mean values of three replicate measurements, and the values were represented by plotting the values of relative viscosity of CT-DNA (η/η_0_)^1/3^ against 1/*R*.

#### 4.5.3 Agarose Electrophoresis Assay

Complexes (2) and (3) were incubated at 37 °C with a concentration of 0.0625 μg/μL pBR322 plasmid DNA at different concentrations expressed as *r*_i_ = complex:DNA (base pair) ratio. The *r*_i_ used was from 0.01 to 0.20, in a total volume of 20 μL. After an incubation period of 24 h, the mobility of the complex-treated pBR322 samples was analyzed by gel electrophoresis at 70 V in Tris/acetate/EDTA buffer. A control of pBR322 was also incubated, and a 1 kb ladder aliquot was loaded into lane 1 of the gel. The gel was stained with an ethidium bromide aqueous solution, and DNA bands were visualized with a UV-transilluminator UVITEC Cambridge UVIDOC HD2 instrument. The studies were performed with 3% (v/v) DMSO as the final solution.

#### 4.5.4 FRET DNA Melting

FRET experiments were performed in 96-well plates and run on an Applied Biosystems QuantumStudio5 RealTime PCR thermocycler equipped with an FAM filter (λ_ex_ = 492 nm; λ_em_ = 516 nm). Fluorolabeled 21-mer hTel21 oligonucleotide, d­[GGG­(TTA­GGG)_3_], and C-KIT1, d­[AGGG­AGGG­CGCT­GGGAG­GAGGG], were purchased from Eurogentec (Belgium) in HPLC purity grade. The FRET probes used were FAM (6-carboxyfluorescein) and TAMRA (6-carboxy-tetramethylrhodamine). The lyophilized strands were first diluted in Milli-Q water to obtain 100 μM stock solutions. Stock solutions were diluted to a concentration of 400 nM in 60 mM potassium cacodylate buffer (pH 7.4) and then annealed to form G4 structures by heating to 95 °C for 5 min, followed by slowly cooling to room temperature ca. 5 h. Stock solutions (1 mM) of the compounds were freshly prepared and further diluted using 60 mM potassium cacodylate to obtain a final concentration of 2 μM. G4 and compound were mixed to achieve a 5:1 stoichiometry of palladium compound:DNA. Experiments were carried out in a 96-well plate with a total volume of 30 μL. The final concentration of the oligonucleotide was 200 nM and 1 μM compound (with a total percentage of DMSO of approximately 0.1%). The thermocycler was set to perform a stepwise increase of 0.3 °C every 30 s, from 25 to 95 °C, and measurements were acquired after each step. To compare different sets of data, FAM emission was normalized (0 to 1). *T*_m_ is defined as the temperature at which the normalized emission is 0.5. Independent experiments were run in triplicate.

### 4.6 Peptide and Protein Interaction Studies

#### 4.6.1 Glutathione (GSH)

Reactions between the title compounds and GSH were also followed by UV–vis spectrophotometry under an inert atmosphere to overcome oxidation of GSH by air oxygen. Samples contained the metal complexes at low concentrations (∼20 μM) and were prepared using 60% (v/v) DMSO/phosphate buffer solution (pH 7.4) to avoid precipitation. The reactants were separated in the pockets of a tandem cuvette, followed by deoxygenation by argon bubbling. The separate solutions were mixed at 1:30 metal complex:GSH molar ratios, and UV–vis spectra were recorded. Argon was continuously passed over the sample during the whole measurement.

#### 4.6.2 Lysozyme

The interaction of complexes (2) and (3) with hen egg white lysozyme (HEWL) was investigated using a 10^–5^ M solution of the protein. Lysozyme is a widely used model protein for studying the binding of potential metallodrugs. The sample preparation for this assay was carried out using the same protocol of the samples for the DNA interactions studies by UV–vis spectrophotometry. The integrity of the enzyme was checked in each experiment, with a comparison of fresh and incubated samples. UV–vis spectra were recorded before and after incubation with the complex (dissolved in 97% 5 mM Tris/3% DMSO) for 24 h at 37 °C. A metal to protein ratio of 3:1 was used for these studies, a ratio that affords similar results to 10:1 and eases the sample preparation. The experimental time-dependent profiles of the spectra were analyzed as pseudo-first-order reactions by plotting the variation of the absorbance as a function of time.

### 4.7 Cytotoxicity and Antibacterial Studies

#### 4.7.1 Cell Lines, Culture Conditions, and *In Vitro* Cytotoxicity Tests

##### 4.7.1.1 Cell Lines and Culture Conditions

Cell culture reagents were obtained from Merck, and plasticware was obtained from Sarstedt. Human colon adenocarcinoma cell line Colo 205 (ATCC CCL-222) and MCF7 (ATCC HTB-22) breast cancer cells were purchased from LGC Promochem. Cancer cells were cultured in RPMI 1640 medium supplemented with 10% heat-inactivated fetal bovine serum, 2 mM l-glutamine, and 1 mM sodium pyruvate and buffered with 100 mM HEPES. The cells were incubated at 37 °C in a 5% CO_2_, 95% air atmosphere and were detached with Trypsin-Versene (EDTA) solution for 5 min at 37 °C.

##### 4.7.1.2 Cytotoxicity Assays

The tested ligands were dissolved in DMSO, and the stock solutions (5 mM) were diluted in complete culture medium. Two-fold serial dilutions of compounds were made in 100 μL of the medium, horizontally. The semiadherent colon adenocarcinoma cells were treated with Trypsin-Versene (EDTA) solution. They were adjusted to a density of 1 × 10^4^ cells in 100 μL of RPMI 1640 medium and were added to each well, with the exception of the medium control wells. The final volume of the wells containing compounds and cells was 200 μL. The plates containing the cells were incubated at 37 °C for 72 h; at the end of the incubation period, 20 μL of MTT solution (5 mg/mL) was added to each well. After incubation at 37 °C for 4 h, 100 μL of sodium dodecyl sulfate solution (10% in 0.01 M HCl) was added to each well, and the plates were further incubated at 37 °C overnight. Cell growth was determined by measuring the optical density (OD) at 540 and 630 nm with a Multiskan EX plate reader (Thermo Labsystems).

Inhibition of the cell growth (expressed as IC_50_: inhibitory concentration that reduces by 50% the growth of the cells exposed to the tested compounds) was determined from the sigmoid curve, where

$$\mathrm{inhibition}=100-\frac{{\mathrm{OD}}_{\mathrm{sample}}-{\mathrm{OD}}_{\mathrm{medium control}}}{{\mathrm{OD}}_{\mathrm{cell control}}-{\mathrm{OD}}_{\mathrm{medium control}}}\times 100$$

values were plotted against the logarithm of compound concentration. Curves for the data obtained on cancer cells were fitted by GraphPad Prism software^49^ using the sigmoidal dose–response model (comparing variable and fixed slopes). The IC_50_ values were always obtained from at least three independent experiments.

#### 4.7.2 Bacterial Cell Culture and Determination of Antibacterial Effect

Antibacterial activity was determined for the title compounds in various Gram-negative and Gram-positive bacterial strains, namely *Klebsiella pneumoniae*, *Escherichia coli* (ATCC 25922), *Staphylococcus aureus* (272123), methicillin-resistant *Staphylococcus aureus* (ATCC 43300), and *Enterococcus faecalis* (ATCC 29212). The stock solutions of the tested compounds were prepared in DMSO at a 5 mM concentration. In parallel, strains were also treated with reference antibiotics, such as tetracycline, gentamicin, and ciprofloxacin (Merck). All stock solutions were diluted in 100 μL of Mueller Hinton Broth, and then two-fold serial dilutions were performed. Then, a 10^–4^ dilution of an overnight bacterial culture in 100 μL of medium was added to each well, except for the medium control wells. The highest concentration of the compounds in the tested samples was 100 μM. The plates were incubated at 37 °C for 18 h. After that, the MIC values were determined by visual inspection.

## Supplementary Material



## Notes

Detailed collection of the NMR spectra for the complexes, infrared spectra of the complexes, additional UV–vis spectra (conditions not included in the main text), electrophoresis of the complexes with pBR223, and MIC data (PDF)
