# Supplementary material for: Palladium(II) Complexes with Noncovalent Interactions with DNA: Solution Speciation Controlled by Solvent Identity, pH, and Concentration
Source: Inorg Chem. 2025 Nov 12;64(46):22852–65. doi: 10.1021/acs.inorgchem.5c04027 (PMC12648656; doi:10.1021/acs.inorgchem.5c04027)
Supplement: Supplementary file 1 [file ic5c04027_si_001.pdf]

Supporting Information

Palladium(II) Complexes with Noncovalent Interactions to DNA. Solution Speciation Controlled by Solvent Identity, pH and Concentration

David Fabra[a], János P. Mészáros,[b] Ana I. Matesanz,[a] Gabriella Spengler,[c] Francisco Aguilar Rico,[a] Guillermo Moreno-Alcántar,[d] Angela Casini,[d] Éva A. Enyedy[b*], Adoracion Gómez Quiroga[a*]

[a] Department of Inorganic Chemistry, (IADCHEM) Universidad Autónoma de Madrid, Calle Francisco Tomás y Valiente, 7, 28049 Madrid, Spain
[b] Department of Molecular and Analytical Chemistry, Interdisciplinary Excellence Centre, University of Szeged, Dóm tér 7-8, H-6720 Szeged, Hungary
[c] Department of Medical Microbiology, Albert Szent-Györgyi Health Center and Albert Szent-Györgyi Medical School, University of Szeged, Semmelweis u. 6, H-6725 Szeged, Hungary
[d] Chair of Medicinal and Bioinorganic Chemistry, School of Natural Sciences, Department of Chemistry, Technische Universität München, Lichtenbergstrasse 4, 85748 Garching b. München, Germany

Corresponding Author

*adoracion.gomez@uam.es, enyedy@chem.u-szeged.hu

Content

Figure S1. 1. ^{13}C NMR spectrum of HL. 2. Aromatic region of the ^{1}H-NMR spectrum of the HL (a) in DMSO, and (b) after the addition of D_2O S3

Figure S2. (A) The suggested deprotonation steps of the ligand (I and II), and the L^- tautomerism (III), (B) pH-dependent UV-vis spectra of HL (C) Absorbance change at 347 nm. (D) Proton dissociation constants of HL as the function of $1/\varepsilon_r$(solvent). {c(HL) = 25.0 μM; I = 0.10 M (KCl); 60% (v/v) DMSO/H_2O; ℓ = 1 cm; t = 25.0 °C} S4

Figure S3. ^{1}H- (top) and ^{13}C NMR (bottom) spectra of complex (1) in DMSO-d_6 .... S5

Figure S4. Detail of the aliphatic region of the 2D [^{1}H, ^{13}C] HMQC NMR spectra of A) complex 1; B) complex 2; and C) complex 3, and d) Complex [PdLCl(DMSO)] spectra freshly prepared from the compound published at Ref 1. In all cases no DMSO peak can be observed between 3.3 and 3.8 ppm, but only at 2.54 ppm. S6

Figure S5. ^{1}H-NMR spectra of complex (2) at 0.5 mM. The insert shows the aliphatic region where no DMSO coordination can be observed S6

Figure S6. Downfield region of the ^{1}H NMR spectra of complex (2): freshly dissolved in DMSO-d_6; protected from light, recorded 1 day after dissolution and upon visible light irradiated. {c(complex 2) ~ 3 mM (peaks suffer minor shift upon irradiation after 1 day and are indicated with ♦, the peaks of complex 3 caused by the concentration are indicated with ✶); t = 25.0 °C} S7

Figure S7. Time dependent UV-Vis spectral changes and photostability of samples a) (2) and c) (3) exposed to visible light for 1 day. b) Continuous spectral change in the first 150 min for complex (2); inset shows the absorbance at 313 nm plotted against the time. {c(complex 2) = 13.4 μM; c(complex 3) = 15.8 μM; solvent: DMSO; ℓ = 1 cm; t = 25.0 °C}. Changes observed in a) may be caused by the light-induced E/Z photoisomerism, a process described for several hydrazones.[2] S7
Figure S8. The three deprotonation processes of complex (2), separated from each other for the sake of clarity. {*c*(complex (2)) = 20.0 μM; *I* = 0.10 M (KCl); 60% (v/v) DMSO/H_2O; ℓ = 1 cm; *t* = 25.0 °C} S8
Figure S9. Time-dependent UV-vis absorption spectra of a) complex 2 in 3% (v/v) DMSO/5 mM Tris buffer b) complex 2 in 60% (v/v) DMSO/5 mM Tris buffer c) complex 3 in 3% (v/v) DMSO/5 mM Tris buffer (pH = 7.4). {*c*(complex 2 and 3) = 20.0 μM; *I* = 0.10 M (KCl); ℓ = 1 cm; *t* = 25.0 °C} S8
Figure S10. Time-dependent UV-vis absorption spectra of a) complex 2 and b) complex 3 in 1% (v/v) DMSO/5 mM Tris buffer. {*c*(complexes 2 and 3) = 20.0 μM; ℓ = 1 cm; *t* = 25.0 °C} 9
Figure S11. Time-dependent UV-vis absorption spectra of a) complex 2 in 60% (v/v) DMSO/5 mM phosphate buffer (pH = 7.4); b) complex 3 in 60% (v/v) DMSO/5 mM phosphate buffer (pH = 7.4). {*c*(complex 2) = 20.0 μM; *I* = 0.10 M (KCl); ℓ = 1 cm; *t* = 25.0 °C} S9
Figure S12. Gel electrophoresis with pBR322 in two conformations (SC and OC). Lane 1: 1 kb DNA ladder; lane 2: pBR322 control; lanes 3–6: complex (2) at r_i: 0.01, 0.05, 0.10 and 0.20; lanes 7-10: complex (3) at r_i: 0.01, 0.05, 0.10 and 0.20; and lanes 11 to 14: cisplatin at r_i: 0.01, 0.05, 0.10 and 0.20. c_{DNA} = 0.0625 μg μL^{-1}........ S9
Figure S13. Example plots of the absorbance variation at 280 nm of the reaction between lysozyme and the palladium complexes 2 and 3 as a function of time. Kinetic calculations as pseudo-first-order reactions........ S10
Table S1. Minimal inhibitory concentration (MIC) for the title compounds and reference antibiotics (MIC values in μM). S11

1.

2.

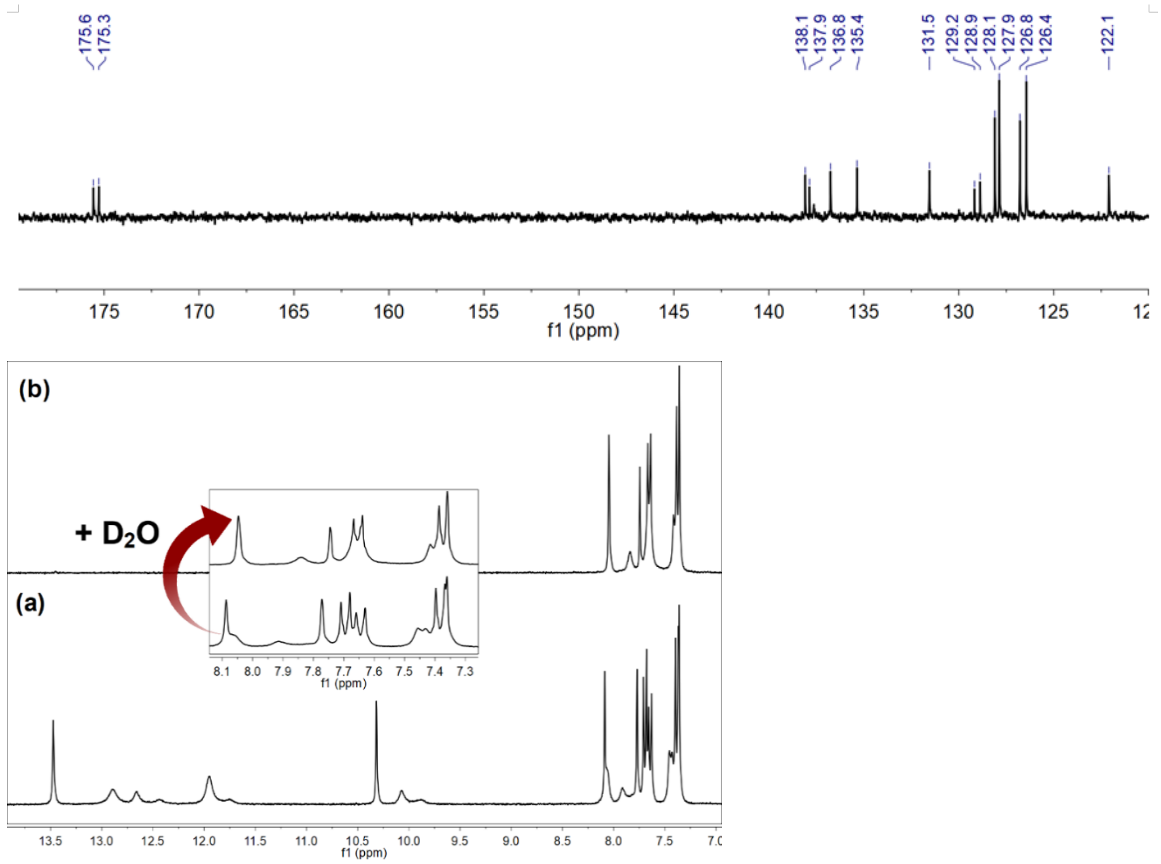

Figure S1. 1. ^{13}C NMR spectrum of HL. 2. Aromatic region of the ^{1}H-NMR spectrum of the HL (a) in DMSO, and (b) after the addition of D_2O.

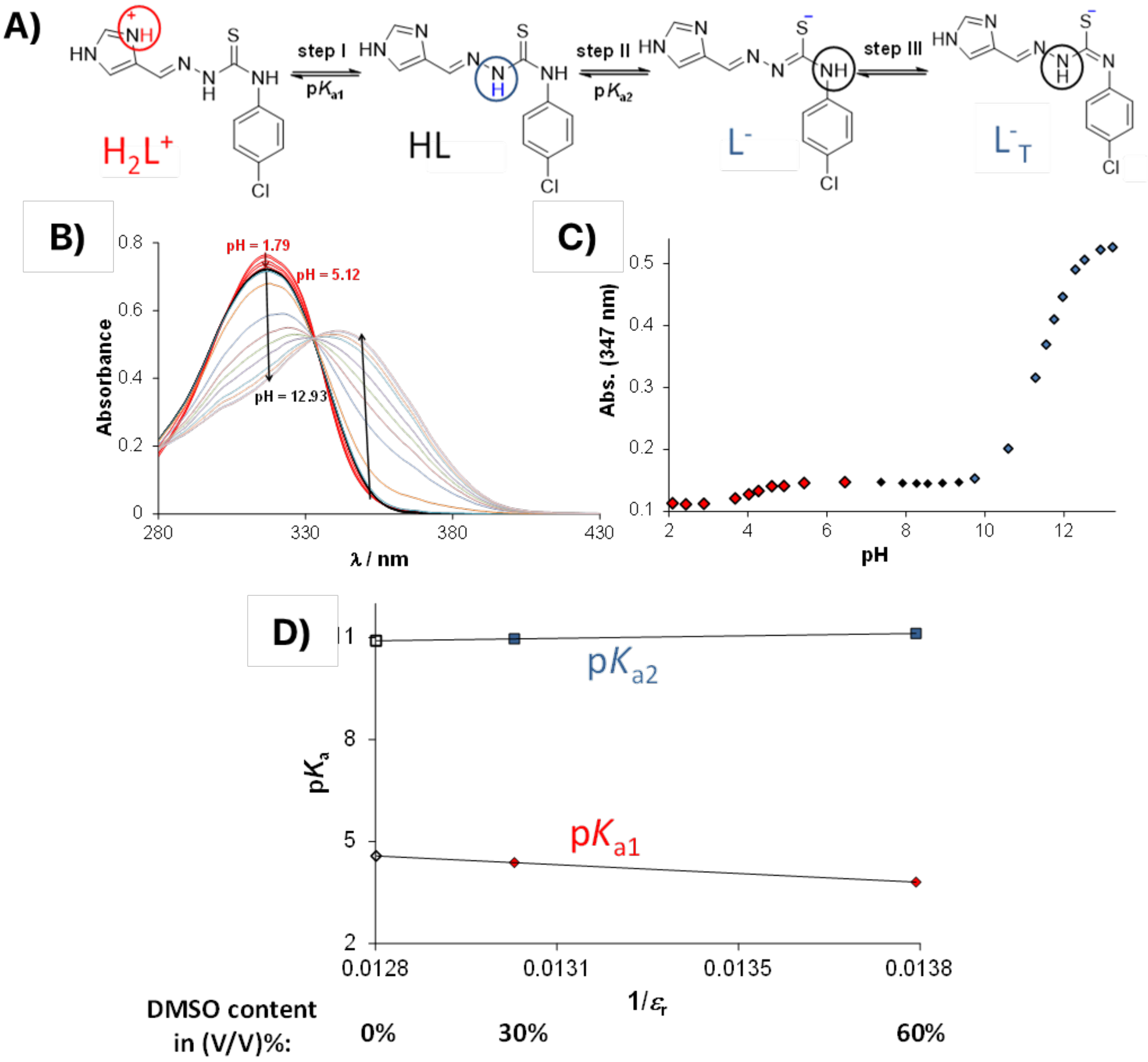

Figure S2. (A) The suggested deprotonation steps of the ligand (I and II), and the L^- tautomerism **(**III), (B) pH-dependent UV-vis spectra of HL (C) Absorbance change at 347 nm. (D) Proton dissociation constants of HL as the function of $1/\varepsilon_r$(solvent). {c(HL) = 25.0 μM; I = 0.10 M (KCl); 60% (v/v) DMSO/H_2O; ℓ = 1 cm; t = 25.0 °C}.

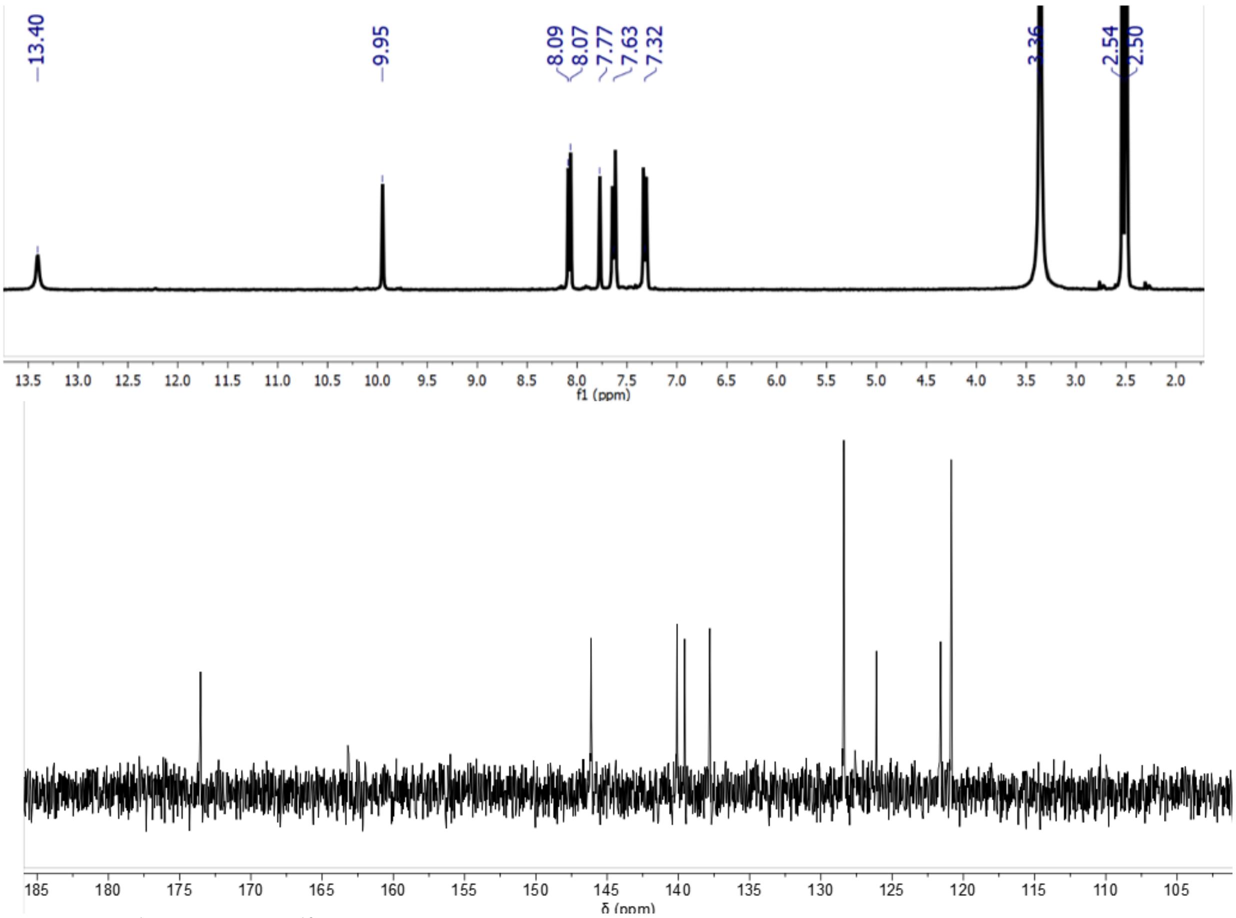

Figure S3. ^{1}H- (top) and ^{13}C NMR (bottom) spectra of complex (1) in DMSO-d_6.

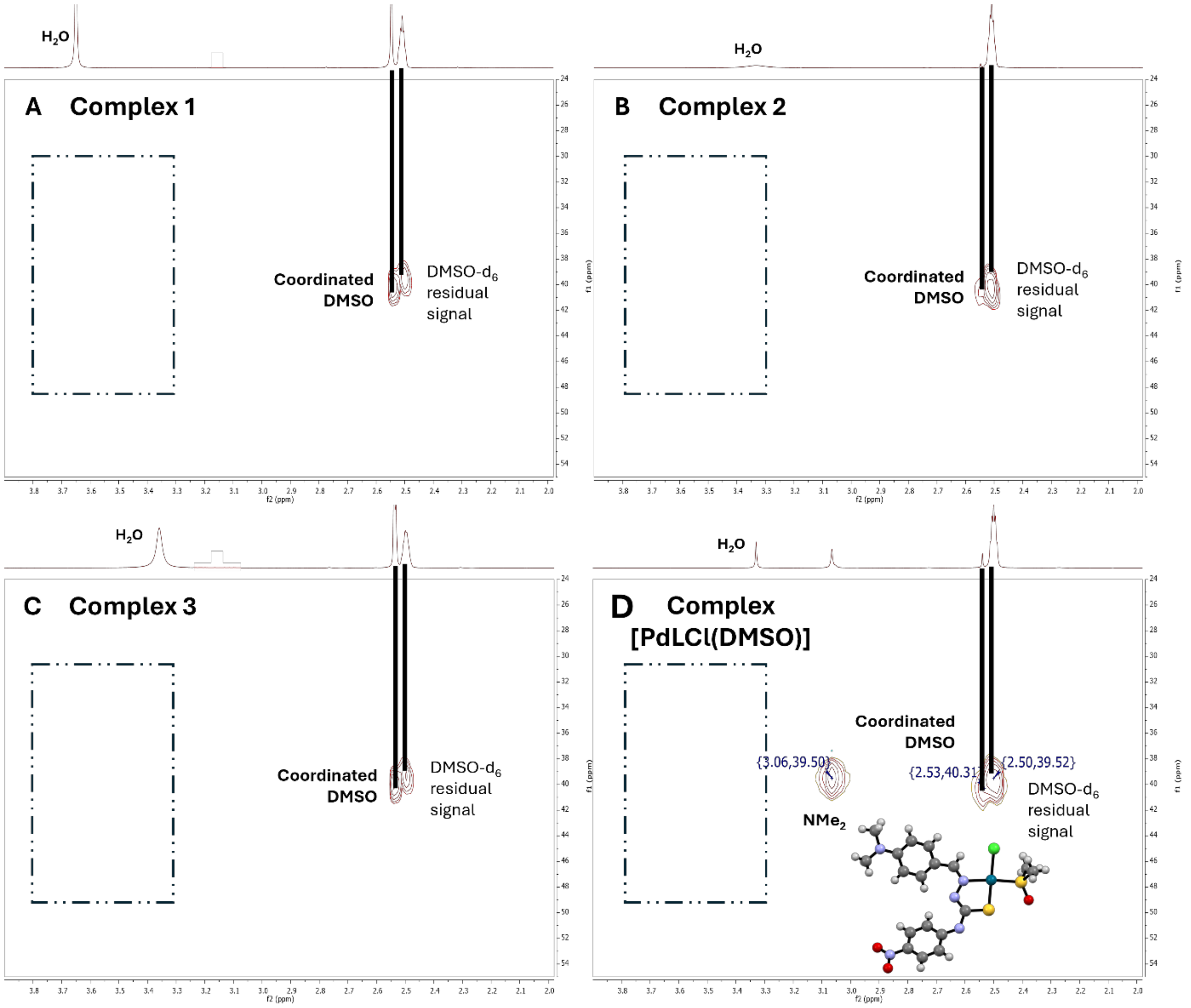

Figure S4. Detail of the aliphatic region of the 2D [^{1}H, ^{13}C] HMQC NMR spectra of A) complex 1; B) complex 2; and C) complex 3, and d) Complex [PdLCl(DMSO)] spectra freshly prepared from the compound published at Ref 1. In all cases no DMSO peak can be observed between 3.3 and 3.8 ppm, but only at 2.54 ppm.

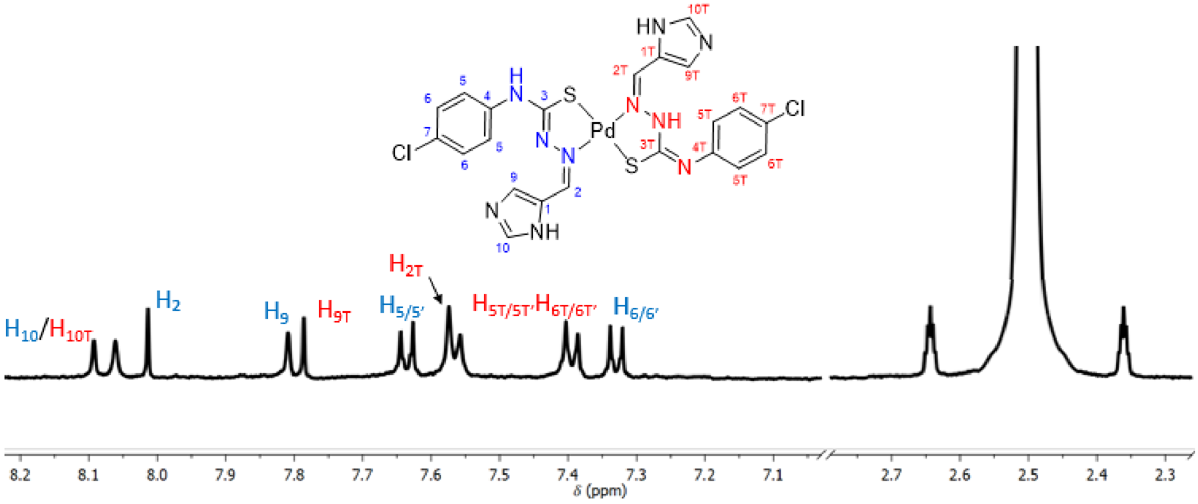

Figure S5. ^{1}H-NMR spectra of complex (2) at 0.5 mM. The insert shows the aliphatic region where no DMSO coordination can be observed.

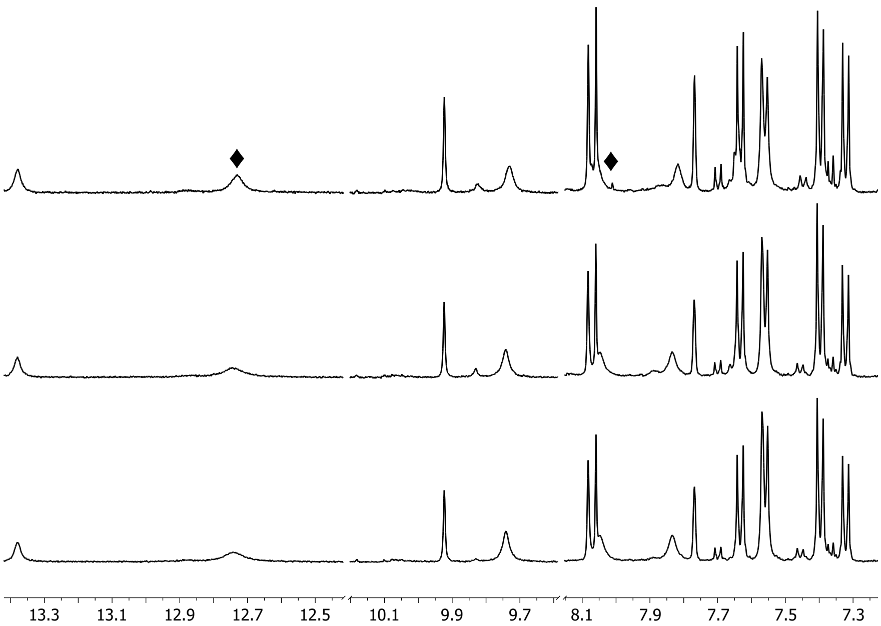

Figure S6. Downfield region of the ^{1}H NMR spectra of complex (2): freshly dissolved in DMSO-d_6; protected from light, recorded 1 day after dissolution and upon visible light irradiated. {c(complex 2) ~ 3 mM (peaks suffer minor shift upon irradiation after 1 day and are indicated with ♦, the peaks of complex 3 caused by the concentration are indicated with ✶); t = 25.0 °C}.

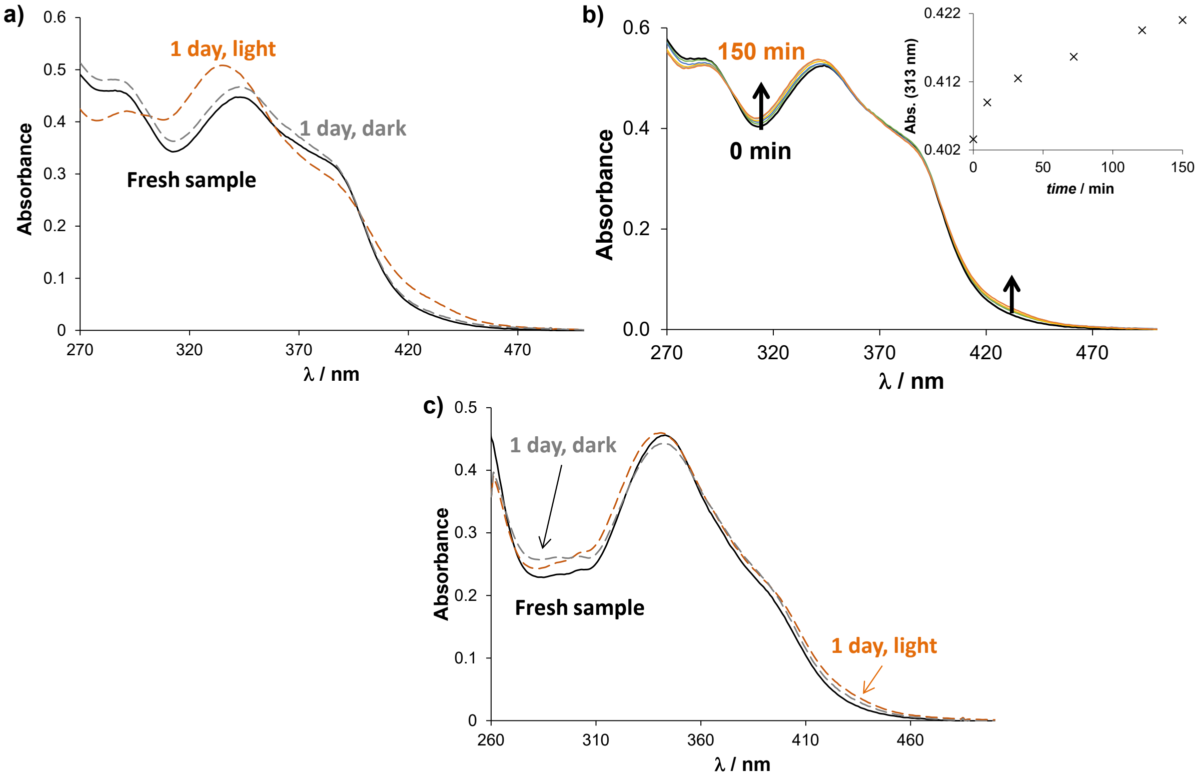

Figure S7. Time dependent UV-Vis spectral changes and photostability of samples a) (2) and c) (3) exposed to visible light for 1 day. b) Continuous spectral change in the first 150 min for complex (2); inset shows the absorbance at 313 nm plotted against the time. {c(complex 2) = 13.4 μM; c(complex 3) =

15.8 µM; solvent: DMSO; ℓ = 1 cm; t = 25.0 °C}. Changes observed in a) may be caused by the light-induced E/Z photoisomerism, a process described for several hydrazones.[2]

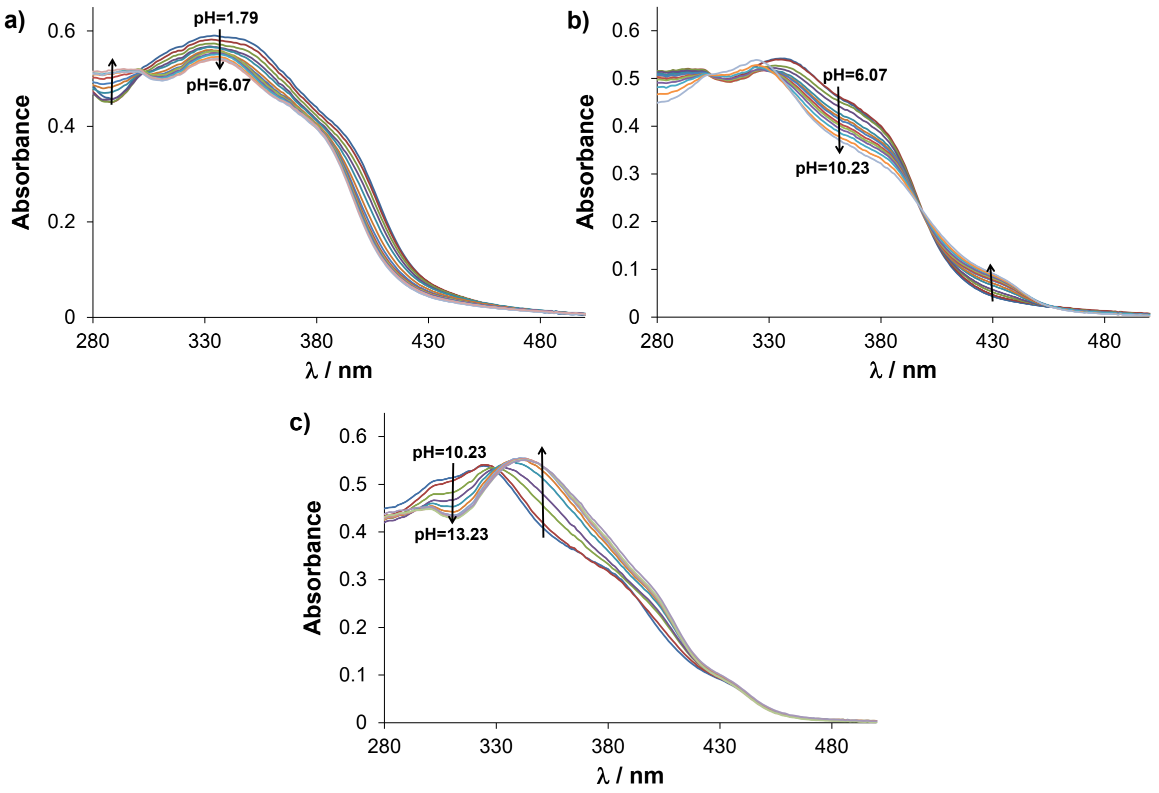

Figure S8. The three deprotonation processes of complex (2), separated from each other for the sake of clarity. {c(complex (2)) = 20.0 µM; I = 0.10 M (KCl); 60% (v/v) DMSO/H_2O; ℓ = 1 cm; t = 25.0 °C}

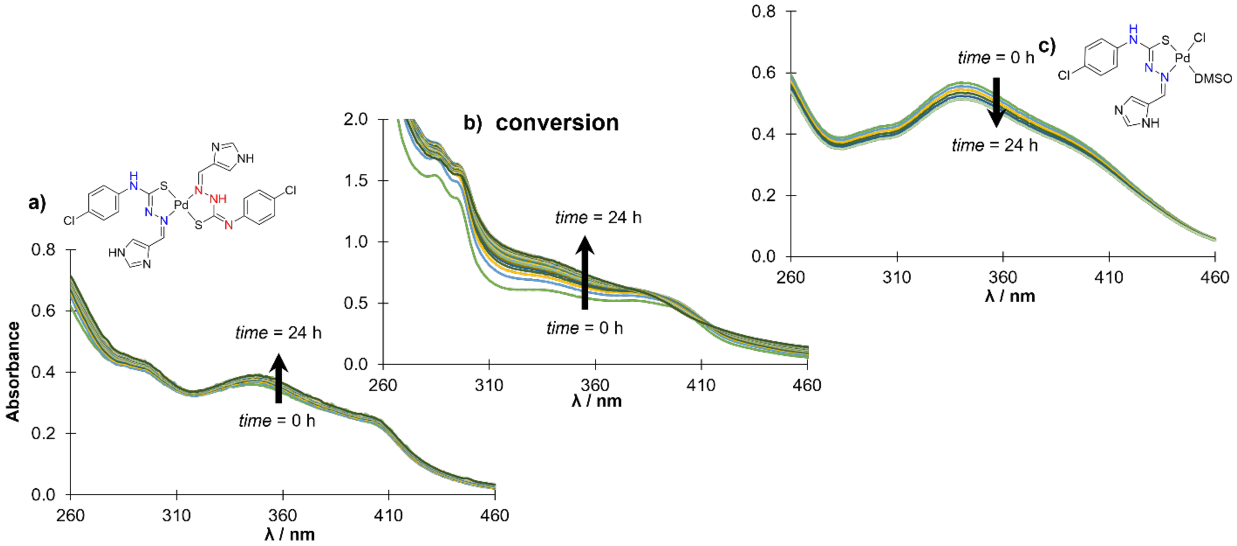

Figure S9. Time-dependent UV-vis absorption spectra of a) complex 2 in 3% (v/v) DMSO/5 mM Tris buffer b) complex 2 in 60% (v/v) DMSO/5 mM Tris buffer c) complex 3 in 3% (v/v) DMSO/5 mM Tris buffer (pH = 7.4). {c(complex 2 and 3) = 20.0 µM; I = 0.10 M (KCl); ℓ = 1 cm; t = 25.0 °C}

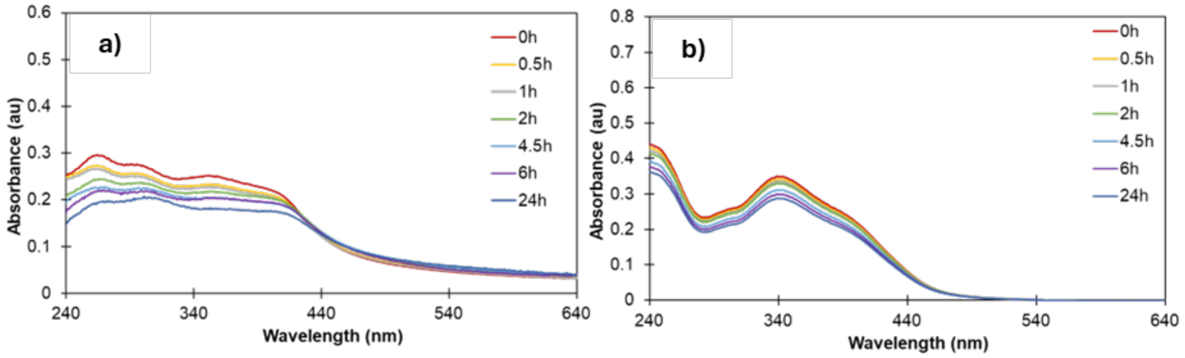

Figure S10. Time-dependent UV-vis absorption spectra of a) complex 2 and b) complex 3 in 1% (v/v) DMSO/5 mM Tris buffer. {c(complexes 2 and 3) = 20.0 µM; ℓ = 1 cm; t = 25.0 °C}

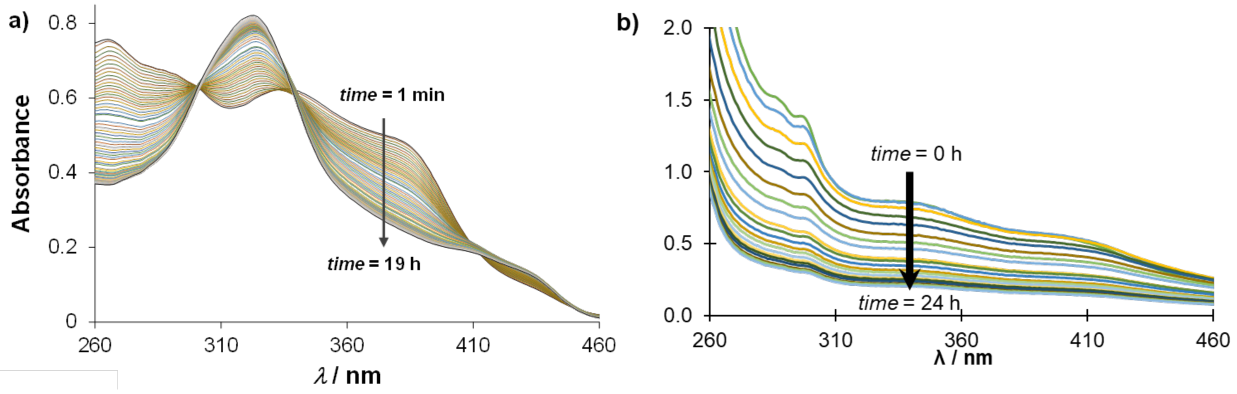

Figure S11. Time-dependent UV-vis absorption spectra of a) complex 2 in 60% (v/v) DMSO/5 mM phosphate buffer (pH = 7.4); b) complex 3 in 60% (v/v) DMSO/5 mM phosphate buffer (pH = 7.4). {c(complex 2) = 20.0 µM; I = 0.10 M (KCl); ℓ = 1 cm; t = 25.0 °C}

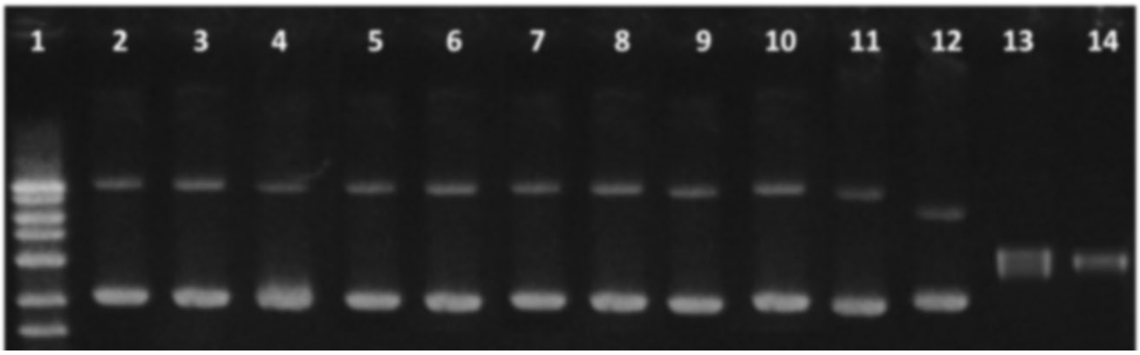

Figure S12. Gel electrophoresis with pBR322 in two conformations (SC and OC). Lane 1: 1 kb DNA ladder; lane 2: pBR322 control; lanes 3–6: complex (2) at r_i: 0.01, 0.05, 0.10 and 0.20; lanes 7-10: complex (3) at r_i: 0.01, 0.05, 0.10 and 0.20; and lanes 11 to 14: cisplatin at r_i: 0.01, 0.05, 0.10 and 0.20. c_{DNA} = 0.0625 µg µL^{-1}.

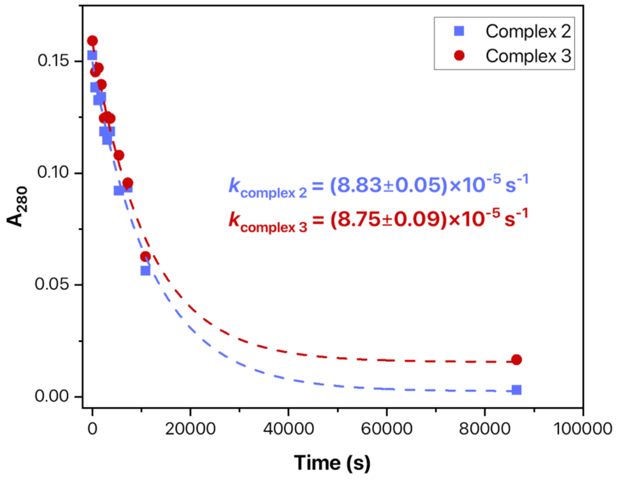

Figure S13. Example plots of the absorbance variation at 280 nm of the reaction between lysozyme and the palladium complexes 2 and 3 as a function of time. Kinetic calculations as pseudo-first-order reactions.

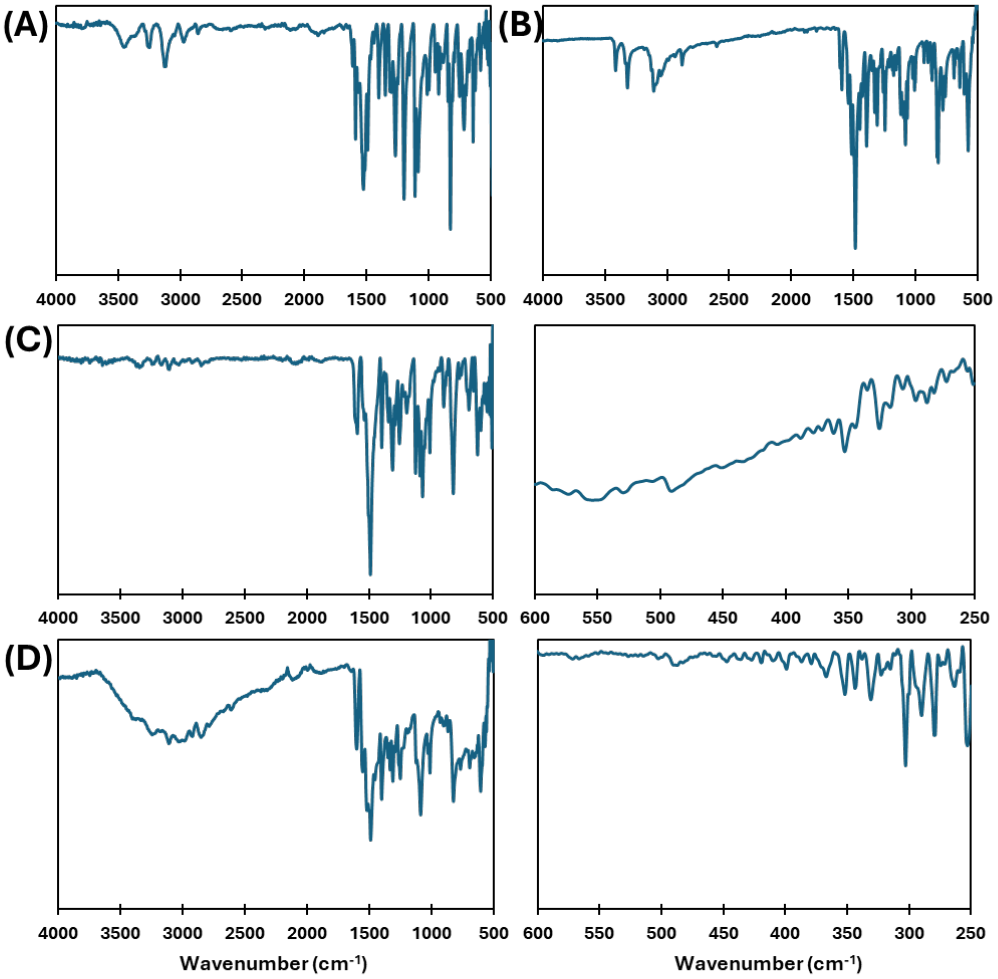

Figure S14. IR spectra of (A) HL, (B) complex 1, (C) complex 2, (D) complex 3.

Table S1. Minimal inhibitory concentration (MIC) for the title compounds and reference antibiotics (MIC values in μM).

	E. coli	*K. pneumoniae*	*E. faecalis*	*S. aureus*	*S. aureus (MRSA)*
HL	25	>100	100	100	50
(2)	>100	>100	>100	>100	>100
(3)	>100	>100	>100	>100	>100
tetracycline	>11.25	2.813	>11.25	>11.25	>11.25
gentamicin	5.235	2.617	10.47	10.47	>10.47
ciprofloxacin	0.2354	0.2354	1.886	1.886	>15.09

References

(1) Hidalgo, T.; Fabra, D.; Allende, R.; Matesanz, A. I.; Horcajada, P.; Biver, T.; Quiroga, A. G. Two Novel Pd Thiosemicarbazone Complexes as Efficient and Selective Antitumoral Drugs. *Inorg. Chem. Front.* **2023**, *10* (7), 1986–1998. https://doi.org/10.1039/D2QI02424A.

(2) Tisovský, P.; Csicsai, K.; Donovalová, J.; Šandrik, R.; Sokolík, R.; Gáplovský, A. Effect of a =X-NH-Fragment, (X = C, N), on Z/E Isomerization and ON/OFF Functionality of Isatin Arylhydrazones, ((Arylamino)Methylene)Indolin-2-Ones and Their Anions. *Molecules* **2020**, *25* (13), 3082. https://doi.org/10.3390/molecules25133082.
